# Genome-Wide Association Study of Topsoil Root System Architecture in Field-Grown Soybean [*Glycine max* (L.) Merr.]

**DOI:** 10.3389/fpls.2020.590179

**Published:** 2021-02-10

**Authors:** Arun Prabhu Dhanapal, Larry M. York, Kasey A. Hames, Felix B. Fritschi

**Affiliations:** ^1^Division of Plant Sciences, University of Missouri, Columbia, MO, United States; ^2^Noble Research Institute, LLC, Ardmore, OK, United States

**Keywords:** genome-wide association study, root system architecture, root complexity score, tap root, lateral root number, lateral root angle, lateral root density, candidate genes

## Abstract

Water and nutrient acquisition is a critical function of plant root systems. Root system architecture (RSA) traits are often complex and controlled by many genes. This is the first genome-wide association study reporting genetic loci for RSA traits for field-grown soybean (*Glycine max*). A collection of 289 soybean genotypes was grown in three environments, root crowns were excavated, and 12 RSA traits assessed. The first two components of a principal component analysis of these 12 traits were used as additional aggregate traits for a total of 14 traits. Marker–trait association for RSA traits were identified using 31,807 single-nucleotide polymorphisms (SNPs) by a genome-wide association analysis. In total, 283 (non-unique) SNPs were significantly associated with one or more of the 14 root traits. Of these, 246 were unique SNPs and 215 SNPs were associated with a single root trait, while 26, four, and one SNPs were associated with two, three, and four root traits, respectively. The 246 SNPs marked 67 loci associated with at least one of the 14 root traits. Seventeen loci on 13 chromosomes were identified by SNPs associated with more than one root trait. Several genes with annotation related to processes that could affect root architecture were identified near these 67 loci. Additional follow-up studies will be needed to confirm the markers and candidate genes identified for RSA traits and to examine the importance of the different root characteristics for soybean productivity under a range of soil and environmental conditions.

## Introduction

Survival and performance of plants depend on efficient exploration of the soil in search for available water and nutrients (Gruber et al., 2013). Quantification of root growth and development requires repetitive measurements during a growing season, and because such measurements are difficult to acquire for field-grown plants, relatively little is known about root systems. Thus, roots are often referred to as the “hidden half” of the plant yet are critically important (Waisel et al., 2002). Root system architecture (RSA) was well-defined by Fitter et al. (1991) and encompasses the spatial arrangement of roots of different ages and classes (Lynch, 1995; Malamy, 2005; Osmont et al., 2007). RSA varies within and among species due to genetics yet also displays phenotypic plasticity in response to the environment (Lynch, 1995). Root system characteristics largely have been ignored by plant breeders and were not targeted as selection criteria as part of the 1960's green revolution. However, understanding RSA holds promise for the discovery, manipulation, and exploitation of root characteristics to both increase plant yield and optimize agricultural land use (Waines and Ehdaie, 2007; Den Herder et al., 2010).

The RSA in *Arabidopsis* and cereals, including its potential for genetic improvement in crops, and high-throughput root phenotyping methodologies are well-reviewed (De Dorlodot et al., 2007; Zhu et al., 2011; Smith and De Smet, 2012; Rich and Watt, 2013). While fundamental RSA is determined by constitutive genetics, substantial modifications are induced by environmental cues (Malamy, 2005). The more dynamic changes in overall RSA through time are deemed root plasticity, which reflects changes in three-dimensional distribution, shape, and branching patterns that may allow the plant to efficiently respond to different environmental conditions (Malamy and Benfey, 1997; Osmont et al., 2007; Péret et al., 2009; Pacheco-Villalobos and Hardtke, 2012).

The soybean [*Glycine max* (L.) Merr.] root system is relatively simple with three distinct morphological components: the primary root, commonly called the taproot, which originates as the radicle from germinating seeds; lateral roots, often referred to as secondary roots that emerge from the taproot; and tertiary and higher-order lateral roots that originate from the secondary and successive order lateral roots (Lersten and Carlson, 2004). The first study of a soybean root system was performed in the 1930's (Borst and Thatcher, 1931). In 1953, it was hypothesized that the root distribution is related to the soil water extraction pattern because the zone of the greatest uptake of the plant root is behind the growing apex of the whole system (Brouwer, 1953), and this was verified in soybeans in the 1970's by studies showing correlations between water uptake and root depth (Allmaras et al., 1975; Arya et al., 1975) and root length density (Burch et al., 1978). Root developmental plasticity, especially morphological and architectural changes, is an adaptive response to avoid plant water deficit and/or nutrient deficiency by exploring a greater soil volume or a given soil volume more thoroughly. Indeed, root developmental plasticity is observed in soybean in response to various conditions, including differences in water and nutrient availabilities (Silvius et al., 1977; Bacanamwo and Purcell, 1999; Zhao et al., 2004).

The intrinsic ability of plant roots to extract water from deeper soil profiles can enable plants to maintain optimal water relations, as well as carbon assimilation, under water-deficit stress (Fenta et al., 2011). One of the major factors influencing soybean rooting depth is the tap root (primary root) elongation rate (Kaspar et al., 1984). Deep taproots, with greater density of lateral roots that increase the surface area for absorption, contribute to drought avoidance in soybean (Ha et al., 2013; Matsuo et al., 2013). The taproot is formed first in soybean and serves as the primary axis for vertical soil exploration. Genotypic variation in tap root elongation rates has been reported (Kaspar et al., 1984), and identification of genotypes with rapidly elongating taproots under water and phosphorus stress conditions may benefit in selection toward high nutrient and water uptake efficient genotypes in soybean (Manavalan et al., 2009; He et al., 2017). While rooting depth is critical in many environments to ensure access to water, the distribution of nutrients in the soil profile is heterogeneous in space and time. For instance, mobile nutrients such as nitrate–nitrogen may readily move into deeper soil layers, whereas immobile nutrients such as phosphorus and potassium often are more abundant in the upper layers of the soil profile (Jobbagy and Jackson, 2001; Ho et al., 2005; Houx and Fritschi, 2011; Lynch, 2013). Consequently, the distribution of roots in the soil in space and time is critical for efficient exploration of soil resources essential for crop growth and development and, to maximize productivity, should be optimized for different soil types and environmental conditions.

Targeted breeding for root systems tailored to different soils and locations requires a thorough understanding of the implications of different RSA types for these environments, the genetics underlying root system characteristics, and the heritabilities of these traits. While genotypic variation in soybean taproot elongation (Kaspar et al., 1984; Manavalan et al., 2015) and lateral root production (Read and Bartlett, 1972) has been demonstrated, only a limited number of studies aimed to elucidate the genetics underlying soybean RSA. These studies primarily have been based on the use of recombinant inbred line (RIL) populations to discover quantitative trait loci (QTLs) for RSA traits. A RIL population developed from a cross between genotypes known to differ in root architecture identified nine QTLs for six root traits under low-phosphorus conditions and four QTLs for four root traits under high-phosphorus conditions (Liang et al., 2010). Five QTLs associated with a visual rating of root system complexity, which they termed “fibrous roots,” was identified in field-grown soybean evaluated in 2 years (Abdel-Hallem et al., 2011), and an another study reported nine QTLs for five root characteristics based on data in 1 year (Brensha et al., 2012). Soybean seedling root traits were studied in a large RIL population grown in a modified hydroponic system and identified five QTLs for maximum root length, four QTLs for lateral root number, three QTLs for root weight, and three QTLs for root volume (Liang et al., 2014). Given the limited information about the genetics underlying soybean root architecture available to date, and the limited number of genotypes upon which this information is based, an examination of topsoil RSA of a diverse population of soybean genotypes can be expected to provide novel insights. Thus, the objectives of this study were to assess a broad range of RSA traits in a soybean diversity panel and to conduct a genome-wide association analysis to identify genomic regions conditioning topsoil root characteristics under field conditions.

## Methods

### Field Locations and Experimental Design

Field experiments were conducted at three locations, namely, Rollins Bottom in Columbia, MO (38°55′37.5″N, 92°20′44.6″W), in 2012 and 2013, and at the Rhodes Farm (Rhodes) near Clarkton, MO (36°48′78.7″N, 89°96′32.8″W), in 2013. At Rollins Bottom, the soil is a Haymond silt loam soil (course-silty, mixed, superactive, mesic Dystric Fluventic Eutrudepts); and at Rhodes, it is a Malden fine sand (mixed, thermic Typic Udipsamments). Seeds of 341 maturity group IV soybean accessions, originally obtained from the USDA Soybean Germplasm Collection, were planted in a randomized complete block design (RCBD) with three replications in each environment. Genotypes were planted at ~2.5-cm depth to a density of 25 seeds m^−2^ in single-row plots measuring 6.1 m (2012) and 2.4 m (2013) in length at Rollins Bottom and 4.6 m in length at Rhodes. Row distance was 0.76 m in all three environments. In 2012 at Rollins Bottom and in 2013 at Rhodes, the experiments were watered with an irrigation gun and a lateral-move irrigation system, respectively. No irrigation was applied at Rollins Bottom in 2013. Pre-plant application of P and K was conducted based on soil test results as recommended by the University of Missouri, Columbia. Weeds were controlled using pre- and post-emergence herbicide applications supplemented by manual weeding as needed. No insecticide applications were conducted. Of the 341 diverse soybean accessions planted, 289 accessions were used for a genome-wide association study (GWAS), while the remaining accessions were excluded because of missing phenotype or marker data. Throughout the remainder of the text, Rollins Bottom 2012 and 2013 experiments and the Rhodes 2013 experiment will be referred to as RB12, RB13, and RH13, respectively.

### Characterization of Topsoil Root System Architecture

Root crown phenotyping was used to determine the RSA of the top portion of the root system, the root crown (York, 2018). When the genotypes reached the beginning seed to seed filling stages, soybean stems were cut a 10–15 cm above the soil surface, and shoots were removed from the row. Root crowns were excavated to a depth of ~0.25 m using a single-row potato digger (SP50, Checchi & Magli, Budrio, Italy) pulled by a tractor. Root systems from five plants were removed from the middle third of each plot and were shaken by hand to remove the remaining loosely attached soil. Root phenotypes were determined using a combination of visual ratings, counting, and measurements as indicated in Table 1. Overall Complexity Score (OCS) was scored visually with the simplest root systems (least fibrous) assigned a score of 1 and the most complex root systems (most fibrous) assigned a score of 5, and intermediate root systems were scored as 2, 3, or 4. A visual scoring system ranging from 1 to 4 was used for the Taproot (TRT) with well-defined, prominent taproots scored as one and missing taproots scored as four. Primary and secondary lateral roots at different positions on the excavated root system were counted manually. Root angles were determined using a plexiglass board made into a 180° protractor with angles indicated every 5°. With the use of this protractor, angles of lateral roots emerging from the taproot (primary lateral roots) were determined based on the position of the lateral root at 5 cm from the point of emergence from the taproot.

**Table 1:** List of root traits measured with their abbreviations and their rating methodology.

**Name of trait**	**Rating methodology or score**
Overall Complexity Score (OCS)	Visual rating of the overall complexity: 1 = very simple, 2 = simple, 3 = average, 4 = complex, 5 = very complex.
Taproot (TRT)	Visual rating of taproot definition: 1 = well-defined, 2 = poorly defined/small, 3 = not defined, 4 = broken/missing.
Upper Primary Lateral Root Number (UPLN)	Number of primary lateral roots emerging from the taproot in the top half of the root crown.
Upper Secondary Lateral Root Density (USLD)	Number of secondary lateral roots in a 2-cm window of one randomly selected upper primary lateral root located at ~2 to 4 cm from the parent root's origin.
Upper Primary Lateral Root Angle Average (ULAA)	Angles of up to five upper first-order lateral roots to the nearest 5°. Angles measured at 5 cm from taproot. Larger diameter roots prioritized for measurements.
Upper Primary Lateral Angle Range (UPAR)	Difference between maximum and minimum angles of upper lateral roots measured for ULAA determination.
Lower Primary Lateral Root Number (LPLN)	Number of primary lateral roots emerging from the taproot in the bottom half of the root crown.
Lower Secondary Lateral Root Density (LSLD)	Number of secondary lateral roots in a 2-cm window of one randomly selected lower primary lateral root located at ~2–4 cm from the parent root's origin.
Lower Primary Lateral Root Angle Average (LLAA)	Angles of up to 5 lower first-order lateral roots to the nearest 5°. Angles measured at 5 cm from taproot. Larger diameter roots prioritized for measurements.
Lower Primary Lateral Root Angle Range (LPAR)	Difference between maximum and minimum angles of lower lateral roots measured for LLAA determination.
Total Number of Primary Lateral Roots (NPL)	Total number of lateral roots emerging from the taproot (UPLN + LPLN).
Average Lateral Density (ALD)	Average of USLD and LSLD.

*Soybean accessions (289) were evaluated at three site-years, namely, at Rollins Bottom in 2012 and 2013 and at Rhodes in 2013*.

### Statistical Analysis

Descriptive statistics were computed for all measured variables using the PROC MEAN procedures of SAS (Sas-Institute-Inc., 2004). To analyze genotype × environment interactions of raw phenotypic means and best linear unbiased predictions (BLUPs), the 2 years at Rollins Bottom and 1 year at Rhodes were treated as three environments, and analysis of variance was performed using the PROC MIXED procedure using the model suggested by Bondari (2003) and Piepho (1994), where the genotype was treated as a fixed effect, and replication nested within environment was treated as a random effect.

BLUPs were used to minimize the effects of environmental variation for a genome-wide association analysis. The phenotypic values of all root traits per genotype were derived within each environment and across all environments using the *lmer* package in *R* (version 3.3.2). Broad-sense heritabilities were determined using a SAS (Sas-Institute-Inc., 2004) program as previously described (Piepho and Möhring, 2007; Dhanapal et al., 2015b).

### Principal Component Analysis, Population Structure (Q), and Kinship Matrix (K)

To better understand the dependencies among traits, Pearson correlation coefficients were computed in *R* (version 3.3.2). Principal component analysis (PCA) was performed using original phenotypic values of all genotypes for all root traits in *R* (version 3.3.2). Data were centered and scaled using the *scale* function, and then the *prcomp* function was used to conduct the PCA. The scores, or rotations, from principal components (PCs) 1 and 2 were selected as aggregate traits to be included in the GWAS.

For the 289 genotypes included in the GWAS analysis, single-nucleotide polymorphism (SNP) markers originated from the application of the Illumina Infinium SoySNP50K iSelect SNP Beadchip (Song et al., 2013). The SNP markers available for the 289 genotypes were filtered to ultimately include 31,708 polymorphic SNPs with a minor allele frequency (MAF) ≥5% in the GWAS analysis. The Bayesian model-based program STRUCTURE 2.3.4 (Pritchard et al., 2000) software was used to infer the population structure (Q) using the 31,708 SNPs. The burn-in iteration and Markov chain Monte Carlo (MCMC) replications after burn-in with an admixture and allele frequencies correlated model was used as described previously (Dhanapal et al., 2015a,b). The population structure analysis was performed with 10 independent iterations with the hypothetical number of subpopulations (*k*) ranging from 1 to 10. All soybean accessions were assigned to a subpopulation based on the optimum *k* (*k* = 8), and the population structure matrix (Q) was generated for further association analyses. TASSEL 5.2.3 software (Bradbury et al., 2007; Buckler et al., 2009) was used to generate the kinship matrix (K) based on “scaled Identity by State (IBS)” similarity matrix (Endelman and Jannink, 2012).

### Genetic Diversity of Germplasm and Linkage Disequilibrium

The 31,708 SNPs with MAF ≥5% along 20 chromosomes were included for genetic diversity analysis and linkage disequilibrium (LD) calculation. Clustering of genotypes was conducted with the cladogram function in TASSEL 5.2 (Bradbury et al., 2007; Buckler et al., 2009) to produce a neighbor-joining (NJ) relationship using parsimony substitution models Newick file. The output Newick file was used as input in TreeDyn 198.3 software (Chevenet et al., 2006) to obtain the final tree.

The calculation of pairwise LD (r^2^) among SNPs and identification of haplotype blocks was based upon SNPs within 1-Mb windows as discussed previously (Dhanapal et al., 2015c). LD plot of SNPs was generated in TASSEL 5.2 using Linkage Disequilibrium function with LD sliding window of 50 SNPs and visualized with LD plot function.

### Genome-Wide Association Studies

A GWAS analysis was performed in the *R* package Genome Association and Prediction Integrated Tool (GAPIT) (Bradbury et al., 2007; Lipka et al., 2012). The model employed for an association analysis was a compressed mixed linear model (CMLM) incorporating the kinship matrix (K) to model random effects and the population structure (Q) estimated by the Bayesian model-based program STRUCTURE to model fixed effects (Pritchard et al., 2000; Bradbury et al., 2007; Zhang et al., 2010; Lipka et al., 2012). Significance of marker–trait associations was assessed by performance of multiple testing using QVALUE R 3.1.0 employing the smoother method (Storey and Tibshirani, 2003; Bass et al., 2015), an extension of the false discovery rate (FDR) method (Benjamini and Hochberg, 1995). Markers with *q*FDR < 0.05 were considered significant (Dhanapal et al., 2015c; Herritt et al., 2016), and all markers that satisfied the multiple testing had –log_10_ *P* ≥ 3.12. The GAPIT package produces an *R*^2^ value that includes contributions from the kinship and population structure in addition to the contribution of the SNP. *R*^2^ has been successfully used as an effective measure to compare models rather than an effective measure of contribution of individual SNPs itself. Therefore, in order to have an *R*^2^ that more reflects the contribution of individual SNPs, the model was run in GAPIT without and with SNP. The difference in *R*^2^ values between the two runs was used as the estimate of the *R*^2^ value of individual SNPs for all putative markers obtained in this study.

### Candidate Genes

To identify genes that may be affecting the RSA phenotypes, a region encompassing a ±0.5-Mb window around each of the putative loci was explored for candidate genes in SoyBase (www.soybase.org) (Grant et al., 2010). For the candidate gene search, the GlymaID from Gmax version 2.0 was used, and only primary predicted protein encoding genes were included in the search. The candidate genes were further narrowed down by gene ontology (GO)-based biological, molecular functions, and cellular component descriptions relevant to root function. PFAM, PANTHER, and KOG descriptions assigned by Phytozome (http://phytozome.jgi.doe.gov/pz/portal.html#!info?alias=Org_Gmax) were also taken into consideration based on previous candidate genes reported for root traits in the literature.

## Results

### Diversity in Root Characteristics Among Genotypes and Environment Effects

**Figure 1:**
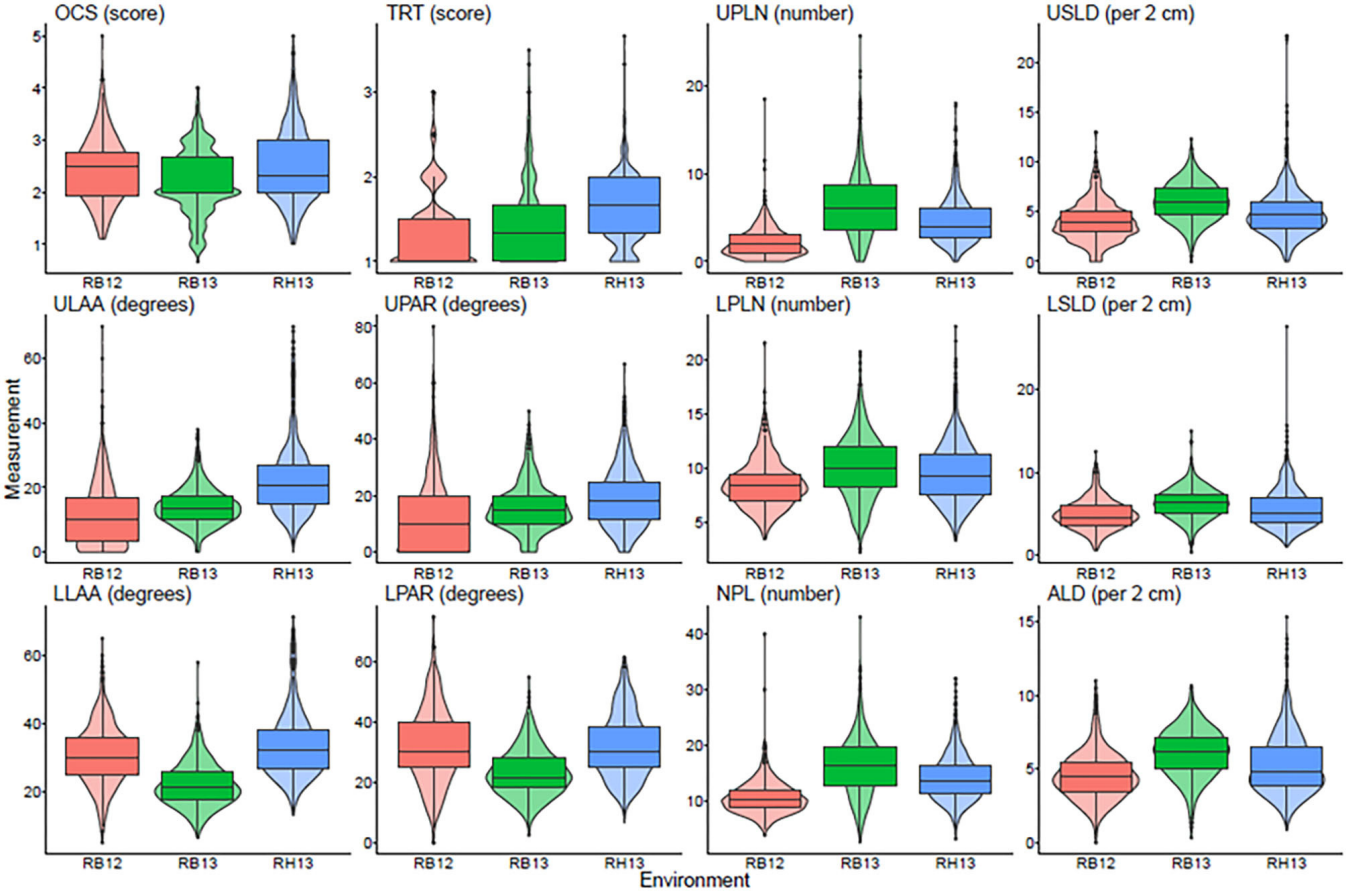
Box plots showing the average and quartiles along with a violin plot giving the continuous distribution within each environment for each measured trait. OCS, Overall Complexity Score; TRT, Taproot; UPLN, Upper Primary Lateral Root Number; USLD, Upper Secondary Lateral Root Density; ULAA, Upper Primary Lateral Root Angle Average; UPAR, Upper Primary Lateral Angle Range; LPLN, Lower Primary Lateral Root Number; LSLD, Lower Secondary Lateral Root Density; LLAA, Lower Primary Lateral Root Angle Average; LPAR, Lower Primary Lateral Root Angle Range; NPL, Total Number of Primary Lateral Roots; ALD, Average Lateral Density. RB12, RB13, and RH13 show distributions of the phenotype values for genotypes within each site-year combination.

A total of 12 root traits were assessed for 289 genotypes in three different environments. A list of these traits and their abbreviations and assessment methodology are provided in Table 1. As Figure 1 and Supplementary Table 1 demonstrate, a broad range of phenotypic values were observed for each trait within each environment, and differences among genotypes were highly significant (*P* < 0.001) for all traits. Additionally, strong environment effects (*P* < 0.001) were observed for all root traits (Supplementary Table 2), but the genotype by environment interactions were significant (*P* < 0.05) only for one half [OCS, UPLN, Upper Primary Lateral Root Number; ULAA, Upper Primary Lateral Root Angle Average; LPLN, Lower Primary Lateral Root Number; LLAA, Lower Primary Lateral Root Angle Average; NPL, Total Number of Primary Lateral Roots] but not the other half [TRT, Taproot; USLD, Upper Secondary Lateral Root Density; UPAR, Upper Primary Lateral Angle Range; LSLD, Lower Secondary Lateral Root Density; LPAR, Lower Primary Lateral Root Angle Range; ALD, Average Lateral Density] of the 12 traits (Supplementary Table 2). The highest mean and median value for OCS were observed in RH13, whereas the lowest mean was observed in RB12 and the lowest median in RB13. Taproot scores ranged from well defined (1) to not defined and absent as indicated by maximum values of 3.0, 3.5, and 3.7 for RB12, RB13, and RH13, respectively. However, as indicated by mean scores <2, most genotypes exhibited defined taproots in all three environments. In some genotypes, upper primary lateral roots were numerous, while in others, they were entirely absent; but on average across all environments and genotypes, 4.5 upper primary lateral roots were observed per plant. Similarly, other traits associated with the UPLN, including USLD, ULAA, and UPAR exhibited large genotypic differences in each environment. The mean genotype USLD across all environments was 5.0 and a maximum range of 0–23 that was found in RH13. The orientation of upper primary lateral roots ranged from horizontal to 70°; and on average across all genotypes, it was the steepest in RH13 (22°), followed by RB13 (14°), and the shallowest in RB12 (11°). Within a plant, the angles of the upper primary lateral roots were the same or very similar in some genotypes, whereas in others, the angles of one root compared with another differed by as much as 80° (UPAR). The mean UPAR was 12° in RB12, 16° in RB13, and 19° in RH13. The mean LPLN was higher in RB13 (10.1) than in RB12 (8.6) and RH13 (9.5), but the range extended up to 23 at RH13. Similarly, mean LSLD was higher in RB13 (6.2) than in RH13 (5.6) and RB12 (4.9). Mean LLAA values were 31° (RB12), 22° (RB13), and 33° (RH13), indicating that lower primary lateral roots generally were steeper than upper primary lateral roots in all three environments. The maximum LPAR observed among the genotypes was the largest in RB12 (75°), and similar in RB13 (53°) and RH13 (55°); but mean LPAR was similar in RB12 (32°) and RH13 (32°), whereas it was lower at RB13 (23°). Among the three environments, both the lowest and highest NPLs were observed in RB13, where the genotype with the lowest NPLs had 2.7 primary lateral roots and the genotype with the highest NPLs had 43.0. Mean NPL was 16.4 in RB13, 14.1 in RH13, and 10.7 in RB12. The same pattern was observed for ALD, where RB12 had the lowest mean (4.5) followed by RH13 (5.2) and RB13 (6.1). More detailed descriptive statistics by environment and across environments are provided in Supplementary Table 1.

### Principal Component and Correlational Analysis Among Root Traits

**Figure 2:**
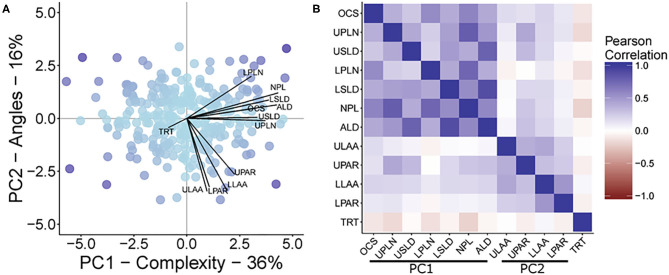
**(A)** A biplot from principal component analysis with principal components 1 and 2, PC1 and PC2, respectively. The points represent the PCA scores, while lines show the magnitude of correlation between each original trait OCS, Overall Complexity Score; UPLN, Upper Primary Lateral Root Number; USLD, Upper Secondary Lateral Root Density; LPLN, Lower Primary Lateral Root Number; LSLD, Lower Secondary Lateral Root Density; NPL, Total Number of Primary Lateral Roots; ALD, Average Lateral Density; ULAA, Upper Primary Lateral Root Angle Average; UPAR, Upper Primary Lateral Angle Range; LLAA, Lower Primary Lateral Root Angle Average; LPAR, Lower Primary Lateral Root Angle Range; TRT, Taproot and the PCA scores. The maximum correlation was 0.86. The color of the points represents the magnitude of the product after multiplying the scores for PC1 and PC2. **(B)** A heat map of the inter-item Pearson correlations of all measured traits. Traits are grouped based on their contribution to the two principal component axes shown in **(A)**.

The first PC (PC1) explained 36.05% of the multivariate variation, with the second PC (PC2) explaining another 15.95% of the variation for a total of 52% of the root trait variation being attributed to these first two PCs (Figure 2). The traits that loaded onto PC1 most strongly were NPL, ALD, LSLD, UPLN, USLD, OCS, and LPLN in descending order of strength. The traits that loaded onto PC2 most strongly were ULAA, LLAA, LPAR, and UPAR in descending order of strength. A correlational analysis among all traits confirmed that traits loading most strongly onto a specific component had greater correlations to other traits loading onto the same component than with traits that loaded onto the other component (Figure 2).

### Genetic Diversity, Population Structure, Linkage Disequilibrium, and Kinship Analysis

The accessions characterized in this study were selected as described in Dhanapal et al. (2015a) and originated from 11 different countries including 187 from South Korea; 46 from China; 30 from Japan; 10 from North Korea; six from Georgia; three from Korea (North or South Korea not recorded in GRIN); three two each from Russia and Taiwan; and one each from India, Mexico, and Romania. The distance-based genetic diversity analysis NJ method identified eight sub-clusters (C1–C8) using model-based subpopulation groups (G1–G8). The genotypes comprising major groups of model-based method were consistent with distance-based methods with few differences (Supplementary Figure 1).

The SNP markers available for the 289 genotypes were filtered to ultimately include 31,708 polymorphic SNPs with a MAF ≥5% for all analyses. The MAF examined with regard to the genotype frequency showed a peak slightly above 10% and then gradually declined to plateau in the range of 20–50% (Supplementary Figure 2a). Among the 20 soybean chromosomes (CHRs), the highest number of SNPs was on CHR 18 (2,622), and the lowest number of SNPs on CHR 20 (1,079) (Supplementary Figure 2b).

An analysis of genetic relatedness using STRUCTURE simulation demonstrated that the calculated average of LnP(D) against *k* = 8 was determined to be the optimum *k*, indicating that eight subpopulations could contain all individuals with the greatest probability. Hence, a *k* value of 8 was selected to describe the genetic structure of the 289 soybean genotypes. The estimated population structure indicated genotypes with partial membership to multiple subpopulations, with few subpopulations exhibiting distinctive identities (Supplementary Figures 2c–e). Significant divergence among subpopulations and average distances (expected heterozygosity) among individuals in the same subpopulations was also assigned (Supplementary Table 3).

An LD analysis was conducted by calculating the square value of correlation coefficient (r^2^) between all pairs of markers (only markers with MAF ≥ 5% were included). Pairwise LD estimates were performed on the complete panel using all 31,708 SNP markers. The average r^2^ of marker pairs suggested that significant LD blocks were observed on several CHRs' regions (Supplementary Figure 3). To demonstrate the difference in LD between euchromatic and heterochromatic regions in the genome, we also compared the average r^2^ values between these regions in each CHR. The LD decay was much higher in the euchromatic compared with heterochromatic regions. In the euchromatic regions, the LD decayed to half of its maximum value within ~80 kb; and in the heterochromatic regions, the LD did not decay to half of the maximum value within 1 Mb (Supplementary Figure 4).

The pairwise kinship estimates based on 31,807 markers showed that most of the pairs of soybean genotypes had zero estimated kinship value. The remaining pair of genotypes suggested some common parental genotypes in their history. Further, the kinship analysis results indicated that most genotypes in the panel have weak kinship, which may be attributed to the broad geographic range of collection of the genotypes and the exclusion of similar genotypes in the analysis. A heat map plot of kinship matrix using average linkage clustering based on SNP markers depicts the existence of different groups among the soybean genotypes (Supplementary Figure 5).

### Heritability and Genome-Wide Association Analysis

The broad-sense heritability was calculated independently for each environment and across all environments. When considering data across all environments, heritability was the greatest for OCS (0.32) followed by LPLN (0.30) and NPL (0.28) (Table 2). Heritabilities calculated for RB13 were the lowest for all root traits. With the exception of TRT across environment, heritabilities within individual environments were equal or lower than heritabilities across all three environments.

**Table 2:** Broad-sense heritability estimates of root system architectural traits OCS, Overall Complexity Score; TRT, Taproot; UPLN, Upper Primary Lateral Root Number; USLD, Upper Secondary Lateral Root Density; ULAA, Upper Primary Lateral Root Angle Average; UPAR, Upper Primary Lateral Angle Range; LPLN, Lower Primary Lateral Root Number; LSLD, Lower Secondary Lateral Root Density; LLAA, Lower Primary Lateral Root Angle Average; LPAR, Lower Primary Lateral Root Angle Range; NPL, Total Number of Primary Lateral Roots; ALD, Average Lateral Density, for 289 soybean accessions evaluated at Rollins Bottom in 2012 and 2013 and at Rhodes in 2013.

**Name of trait**	**RB12**	**RB13**	**RH13**	**Across**
OCS	0.40	0.22	0.60	0.32
TRT	0.14	0.11	0.12	0.10
UPLN	0.37	0.12	0.56	0.17
USLD	0.19	0.10	0.24	0.10
ULAA	0.28	0.11	0.30	0.11
UPAR	0.17	0.11	0.19	0.12
LPLN	0.28	0.14	0.52	0.30
LSLD	0.30	0.12	0.33	0.14
LLAA	0.34	0.13	0.45	0.13
LPAR	0.13	0.11	0.15	0.11
NPL	0.39	0.11	0.59	0.28
ALD	0.28	0.12	0.33	0.18

Genome-wide association analysis across environments identified both unique SNPs (SNPs that are associated with one trait only) and SNPs common among root traits as well as for PC1 and PC2 (Table 3; Supplementary Table 4). A total of 283 (non-unique) SNPs were significantly associated with one or more of the 14 root traits including PC1 and PC2. Of these, 246 were unique SNPs (Table 3) and 215 SNPs were associated with a single root trait, while 26, four, and one SNPs were associated with two, three, and four root traits, respectively. The number of significant SNP associations per root trait ranged from eight for ULAA to 33 for USLD. Considering the co-location of SNPs, the number of putative loci identified for the different root traits ranged from 3 for LPLN to 13 for UPAR (Table 3). For individual root traits, the maximum number of SNPs tagging the same putative locus ranged from 3 (ULAA, UPAR) to 16 (LPAR), but for all root traits, at least one locus was identified by only a single SNP at the threshold used in the study. The maximum *R*^2^ for the SNPs associated with each trait ranged from 0.04 for LLAA to 0.08 for USLD.

**Table 3:** Number of unique single-nucleotide polymorphisms (SNPs), number of putative loci, maximum number of unique SNPs tagging a locus, and the greatest *R*^2^ value observed among the SNPs identified for each root trait (maximum *R*^2^).

**Root traits**	**Unique SNPs**	**Number of putative loci**	**Maximum no. unique SNPs per locus**	**Maximum *R^**2**^***
OCS	18	8	7	0.07
TRT	25	10	4	0.06
UPLN	28	11	9	0.05
USLD	33	9	12	0.08
ULAA	8	5	3	0.05
UPAR	17	13	3	0.05
LPLN	10	3	4	0.05
LSLD	19	8	7	0.05
LLAA	32	8	13	0.04
LPAR	29	8	16	0.06
NPL	16	9	8	0.06
ALD	15	9	4	0.05
PC1	17	11	4	0.06
PC2	16	8	5	0.05

*OCS, Overall Complexity Score; TRT, Taproot; UPLN, Upper Primary Lateral Root Number; USLD, Upper Secondary Lateral Root Density; ULAA, Upper Primary Lateral Root Angle Average; UPAR, Upper Primary Lateral Angle Range; LPLN, Lower Primary Lateral Root Number; LSLD, Lower Secondary Lateral Root Density; LLAA, Lower Primary Lateral Root Angle Average; LPAR, Lower Primary Lateral Root Angle Range; NPL, Total Number of Primary Lateral Roots; ALD, Average Lateral Density, for PC1 and PC2*.

**Figure 3:**
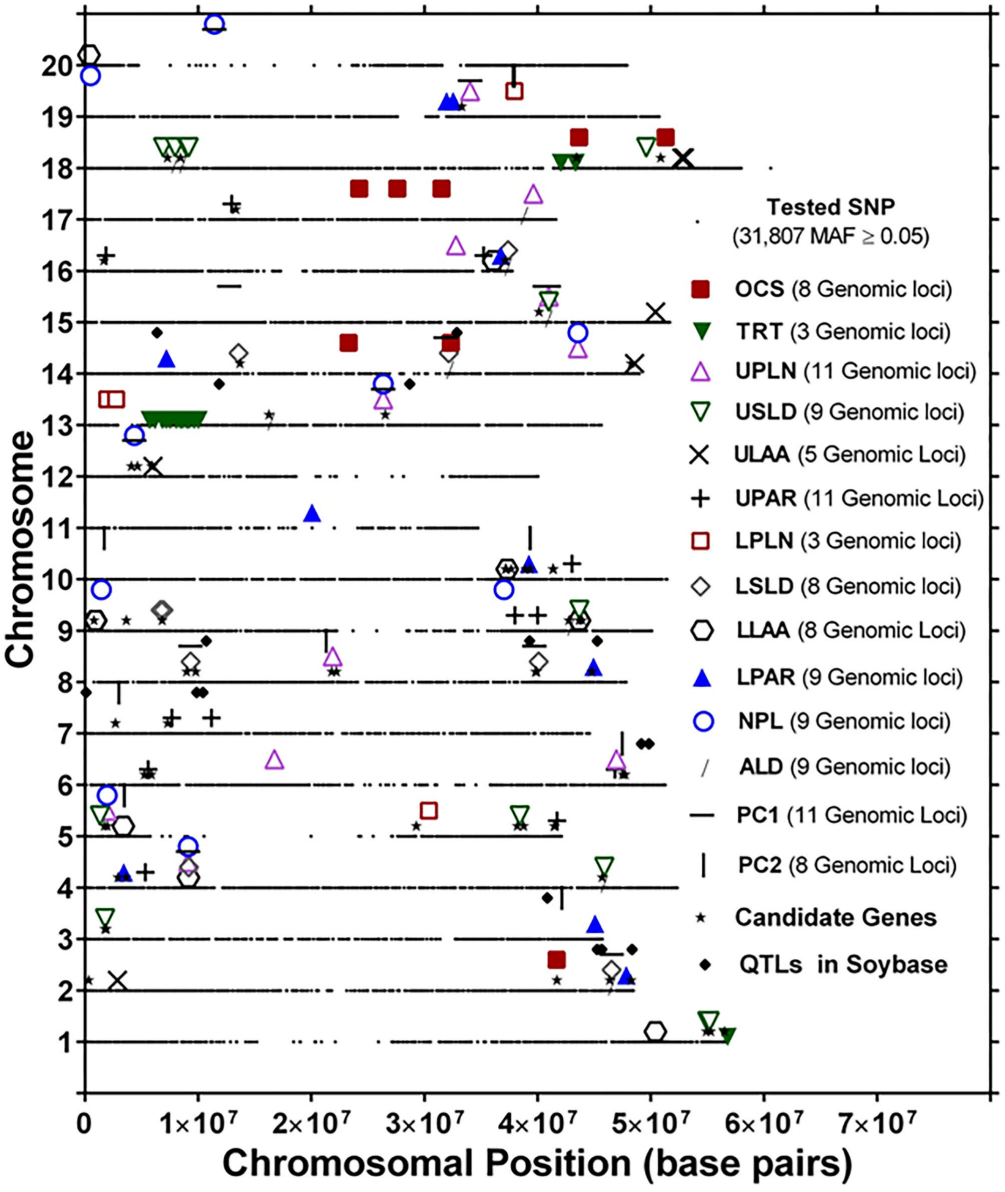
Genomic locations of single-nucleotide polymorphism (SNP) markers showing significant marker–trait associations of root system architecture traits [OCS, Overall Complexity Score; TRT, Taproot; UPLN, Upper Primary Lateral Root Number; USLD, Upper Secondary Lateral Root Density; ULAA, Upper Primary Lateral Root Angle Average; UPAR, Upper Primary Lateral Angle Range; LPLN, Lower Primary Lateral Root Number; LSLD, Lower Secondary Lateral Root Density; LLAA, Lower Primary Lateral Root Angle Average; LPAR, Lower Primary Lateral Root Angle Range; NPL, Total Number of Primary Lateral Roots; ALD, Average Lateral Density]. For each chromosome, the black dots represent the location of SNPs evaluated for association with different root traits. Also shown are the locations of candidate genes identified based on haplotype analysis of significant markers and quantitative trait loci (QTLs) in SoyBase.

**Figure 4:**
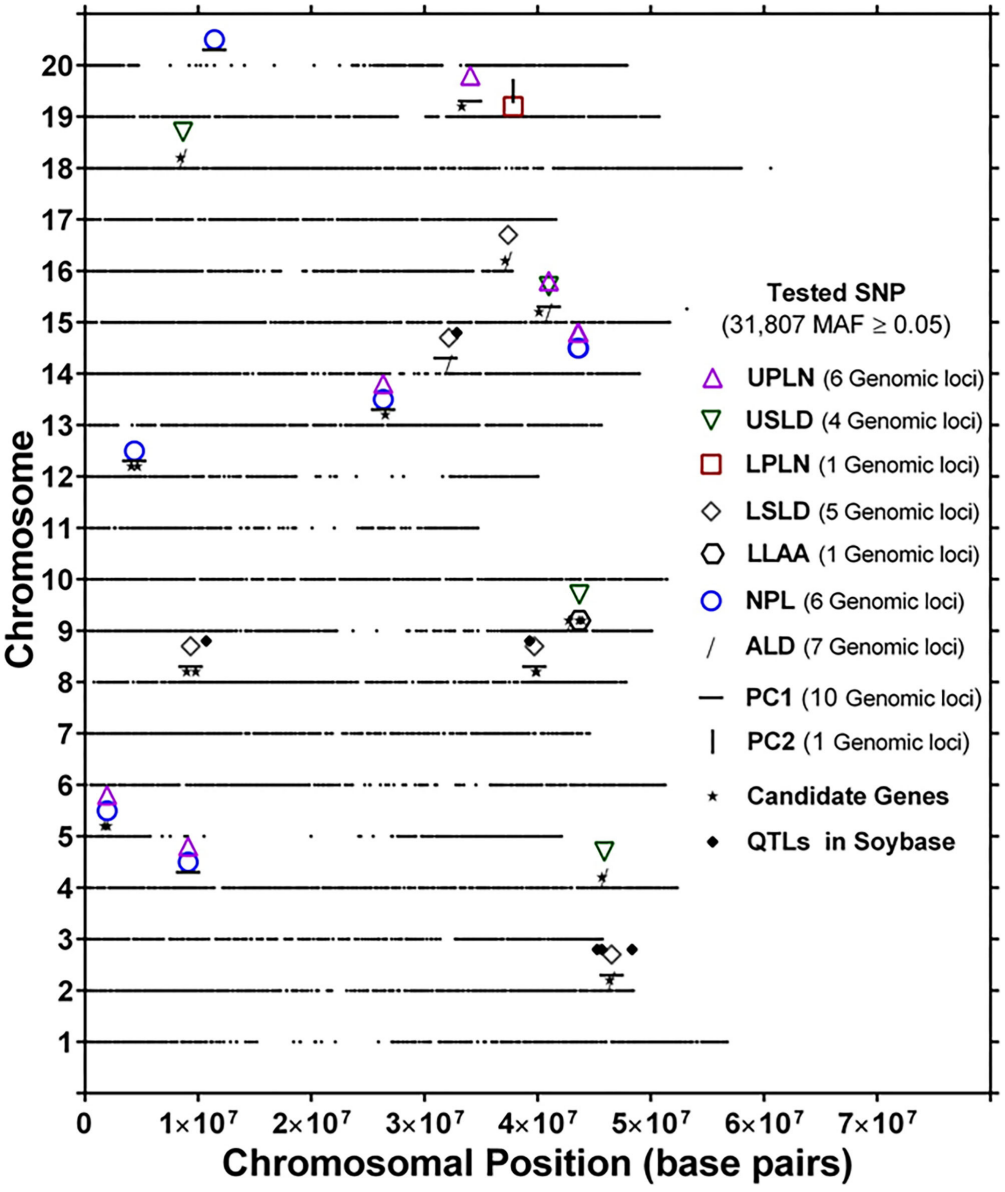
Genomic location of identical single-nucleotide polymorphism (SNP) markers showing significant marker–trait associations for more than one root trait [UPLN, Upper Primary Lateral Root Number; USLD, Upper Secondary Lateral Root Density; LPLN, Lower Primary Lateral Root Number; LSLD, Lower Secondary Lateral Root Density; LLAA, Lower Primary Lateral Root Angle Average; NPL, Total Number of Primary Lateral Roots; ALD, Average Lateral Density; NPL, Total Number of Primary Lateral Roots, for PC1 and PC2]. For each chromosome, the black dots represent the location of an SNP evaluated for association with different root traits. Also shown are the locations of candidate genes identified based on haplotype analysis of significant markers and quantitative trait loci (QTLs) in SoyBase.

The genomic locations of significant SNPs marking putative loci for the different root traits are indicated in Figure 3. Quantile-quantile (QQ) plots and Manhattan plots for different root traits with significant marker–trait associations are shown in Supplementary Figure 6 and Supplementary Figure 7. Specific data for the putative loci and individual SNPs visualized in Figure 3 and summarized in Table 3 are included in Supplementary Table 4. For each root trait, putative loci were identified on three or more CHRs, and several of the putative loci were located near previously identified QTLs (Figure 3). Every CHR had at least one putative locus for one root trait, and several CHRs contained multiple putative loci for different root traits. Indeed, not only did several genomic regions encompass putative loci for several root traits, but 31 SNPs were significantly associated with more than one root trait (Supplementary Table 5). The genomic locations of these multi-root trait “hot spots” are visualized in Figure 4, which shows putative loci marked by both single SNPs associated with multiple traits and putative loci marked by multiple SNPs associated with the same combination of root traits. Each of the 12 root traits and PC1 and PC2 was associated with an SNP that was also associated with at least one other root trait. In total, 13 different combinations of root traits were significantly associated with individual SNPs (Table 4, Figure 4).

**Table 4:** Summary of root trait combinations identified by individual single-nucleotide polymorphisms (SNPs).

	**Root trait combinations**	**SNPs**	**CHR**	**Position**	**Number of SNPs per combination**	**Locus number for combination**
1	LSLD, ALD, PC1	ss715583303	2	46530067	2	1
		ss715618168	14	31978272		2
2	USLD, ALD	ss715588242	4	45895882	2	1
		ss715604322	9	42951837		2
		ss715632710	18	7954314	3	3
		ss715632713	18	7963930		3
		ss715632806	18	8660316		3
3	UPLN, NPL, PC1	ss715589583	4	9104210	2	1
		ss715614264	13	26344379		2
4	UPLN, NPL	ss715590397	5	1960856	9	1
		ss715618902	14	43548260		2
		ss715618903	14	43557868		2
		ss715618905	14	43561539		2
		ss715618908	14	43572822		2
		ss715618911	14	43575830		2
		ss715618915	14	43597753		2
		ss715618917	14	43604766		2
		ss715618918	14	43606363		2
5	LSLD, PC1	ss715602853	8	9332121	2	1
		ss715601637	8	39714639		2
6	USLD, LLAA	ss715604431	9	43681624	1	1
7	NPL, PC1	ss715613088	12	4364475	1	1
8	ALD_PC1	ss715618165	14	31869835	1	1
9	LSLD, ALD	ss715618173	14	32148722	1	1
		ss715624967	16	37323949	2	2
		ss715624973	16	37381270		2
10	UPLN, PC1	ss715634102	19	34015909	2	1
		ss715634106	19	34038285		2
11	UPLN, USLD, ALD, PC1	ss715621832	15	40976845	1	1
12	LPLN, PC2	ss715634639	19	37841812	1	1
13	NPL, PC1	ss715636802	20	11434953	1	1

*UPLN, Upper Primary Lateral Root Number; USLD, Upper Secondary Lateral Root Density; LPLN, Lower Primary Lateral Root Number; LSLD, Lower Secondary Lateral Root Density; LLAA, Lower Primary Lateral Root Angle Average; NPL, Total Number of Primary Lateral Roots; ALD, Average Lateral Density, for PC1 and PC2*.

In some instances, more than one SNP was significantly associated with a particular combination of root traits. Of the 13 different root trait combinations, three root traits, namely, UPLN (NPL, PC1, USLD, and ALD), USLD (ALD, LLAA, UPLN, and PC1), and ALD (LSLD, USLD, UPLN, and PC1), were identified by SNPs tagging four other root traits. Additionally, LSLD (ALD and PC1) and NPL (UPLN and PC1) were marked by SNPs that also were associated with two other root traits. Not surprisingly, PC1 co-localized with multiple root characteristics (ALD, LSLD, UPLN, NPL, and USLD). In contrast, PC2 co-localized with only one trait (LPLN). One of the SNPs (located on CHR 15) associated with PC1 was also associated with three other traits, making it the single SNP associated with the largest number of traits (UPLN, USLD, ALD, and PC1). On CHR 14, one putative locus was marked by eight SNPs associated with both UPLN and NPL, and the other locus was associated with three SNPs that each marked ALD in combination with either LSLD or PC1. The putative locus on CHR 18 was marked by three SNPs that were associated with USLD and ALD. A locus on CHR 16 was marked by two SNPs that were associated with both LSLD and ALD, and a locus on CHR 19 was marked by two SNPs associated with UPLN and PC1. Additionally, the putative locus on CHR 9 was marked by one SNP associated with USLD and ALD and another SNP associated with USLD and LLAA.

### Candidate Genes and Gene Ontology Enrichment Analysis

Putative candidate genes were identified within ±0.5 Mb for all significant SNPs showing marker–trait association for all root traits. Across the 14 traits, 70 candidate genes that may underlie associations with root characteristics were identified (Supplementary Table 6). Among the 70 candidate genes, 20 candidate genes (Supplementary Table 7) were identified near 12 of 17 putative loci associated with more than one root trait. For all 70 candidate genes identified in this study, a GO enrichment analysis was conducted in SoyBase (www.soybase.org; Morales et al., 2013) using the gene model and data mining and analysis option. All 70 genes identified had one or more GOs. The submitted genes could be classified into three overarching GO categories: (i) biological processes including, carbohydrate, lipid metabolic process, signal transduction, embryo, postembryonic and flower development, cell growth, and cellular development process; (ii) cellular component or compartments such as extracellular region, cell wall, intracellular, nucleus, nucleolus, cytoplasm, mitochondria, endosome, golgi apparatus, cytosol, plasma membrane, plastid, and membrane; and (iii) molecular function such as nucleotide, protein and carbohydrate binding, sequence-specific DNA binding transcription factor (TF), and catalytic and transferase activities.

## Discussion

### Descriptive Statistics and Correlations

This study employed root crown phenotyping of a soybean diversity panel in three field environments to assess 14 RSA traits including lateral root numbers, secondary lateral root densities, and lateral root angles (Table 1) and the first two PCs from a PC analysis. Broad ranges of phenotypic values were observed for different RSA traits. Differences between the Rollins Bottom and the Rhodes locations include latitude, soil type, and irrigation (watered with an irrigation gun at RB12, a lateral-move irrigation system at RH13, and no irrigation at RB13), all of which may affect the growth response of soybean. These factors, as well as differences in temperatures and precipitation amounts and distributions, likely modulated the RSA phenotypes. A correlational analysis indicated that numbers and densities of roots tended to be more strongly associated with each other and complexity and that various measures of angles formed a separate co-correlated group.

### Root System Architecture and Their Relationship Among Root Traits

To our knowledge, this is the first use of PCs in GWAS of root traits. PC1 was heavily influenced by “complexity” traits such as the numbers of primary and secondary laterals, while PC2 was more influenced by all the measures of angle. In fact, this structure agrees with earlier reports in maize where nodal root number and lateral root density influenced PC1, while the angles of various nodes of crown roots influenced PC2 (York and Lynch, 2015), which might imply a common data structure among genotypes of all plant species since maize is a monocot and soybean is a dicot. Since data are scaled for PCA, loadings of “complexity” traits onto PC1 indicate that there was generally more variation for these traits than for angle-related traits, which may have implications for breeding, which requires selection gradients. However, these complexity traits are also expected to be more affected by plant allometry (larger plants have more roots intrinsically), which means angles are suitable as traits that are independent from plant size. The discovery of QTLs responsible for both PC1 and PC2 implies a common genetic and developmental basis for the multitude of underlying and correlated traits that form the components. Not surprisingly, PC1 co-localized more often with other traits than any other traits, and only with traits that strongly loaded onto the component. Previous work substantiates that PC scores have greater statistical power for SNP associations than univariate measures (Galesloot et al., 2014). The PCA results were supported by a correlational analysis where traits loading more strongly onto a component were more likely to have stronger correlations with each other than with traits loading onto the other component. PCs of root traits may serve as useful aggregate traits for physiological experimentation, genetic mapping, and breeding programs. The relationships among traits are driven by latent constructs represented by the PCs due to developmental and genetic constraints, and further consideration of these latent constructs will be needed for the future of phenomics (York, 2019).

### Root System Architecture Trait Single-Nucleotide Polymorphisms and Loci

The genome-wide association analysis revealed significant SNPs for all 14 root traits, and each trait was associated with eight to 33 SNPs, which marked three to 13 putative loci per trait (Table 3). Thirty-one SNPs were associated with combinations of two to four traits (Tables 3, 4; Supplementary Table 5). On the one hand, these SNPs may be of particular interest in that they may mark genomic hotspots or key regulatory elements for root architecture but, on the other hand, may be associated with two or more traits because these traits are not independent of each other. Clearly, the 67 loci identified for the 14 root traits indicate that allelic variation exists for each of these traits, which may be explored to alter soybean root system characteristics through breeding. That said, the generally rather low heritabilities, ranging from 10 to 60% based on individual environments and from 10 to 32% when estimated across all three environments, indicate a strong environmental influence on these traits. Given the complexity of phenotyping of root systems of field grown plants, the relatively low heritabilities, in part, could also be a reflection of the phenotyping challenges. Previous reports of heritabilities of soybean root traits based on experiments with RIL populations generally were higher than those found here. Root characteristics examined in soybean grown under low P and high P in the field reported heritabilities of root traits that ranged from 47 to 72% (Liang et al., 2010). Similarly, soybean root characteristics assessed in response to P availability under field conditions have reported heritabilities between 50 and 91% (Ao et al., 2010), and another study reported heritabilities for root hair density and root hair length ranging from 27 to 34% and from 54 to 61%, respectively (Wang et al., 2004).

To date, specific mechanisms underlying root architecture traits growth and developmental process are often unclear. Targeted improvement of root characteristics for specific production challenges requires a thorough understanding of the value and tradeoffs associated with specific root traits and combinations of root traits under particular soil and environmental conditions. However, for the most part, a significant knowledge gap remains about such root trait benefits and tradeoffs. Nevertheless, if beneficial root properties can be determined, the responsible alleles could be bred into elite cultivars using marker assisted breeding. Recently, steeper root angles were observed in drought-tolerant lines of soybean (Fenta et al., 2014), which confirms work in the common bean (*Phaseolus vulgaris*), which has a root system similar to that of soybean (Ho et al., 2004, 2005). At the same time, shallow root angles were shown to improve phosphorus uptake in soybean and in the common bean (Liao et al., 2004). Interestingly, during combined water and phosphorus stress, shallow-angled common bean root systems performed better than steep-angled root systems because the enhanced early P nutrition allowed greater plant growth and deeper rooting (Ho et al., 2005). Due to such tradeoffs and possibly non-intuitive trait integration (York et al., 2013), studies of the functional implications for soil resource acquisition and crop performance are imperative to successfully utilize root traits in breeding programs.

### Relationships of Identified Loci With Previously Reported Quantitative Trait Loci for Root Traits

A literature search for previously identified QTLs for different soybean root traits and SoyBase (https://www.soybase.org/search/previous/index.php) based search using term “root” revealed 19 QTLs that were located within ±0.5 Mb of at least one of two flanking markers close to the putative loci identified in this study. The putative locus identified for LSLD, ALD, LPAR, and PC1 on CHR 2 was located near one QTL previously identified for lateral root number (q*LRN1*-*D1b*-*1*) (Liang et al., 2014) and a QTL (*rdwlpD1b*+W-05) for root dry weight of seedlings grown at low-phosphorus availability (Zhang et al., 2009). A previously identified QTL for root dry weight (*rdwnpA2-05*) at high-phosphorus availability (Zhang et al., 2009) was near a putative locus identified for LSLD and PC1 on CHR 8. Another QTL for root weight (q*RW2*-*f_1*-*1*) identified previously (Liang et al., 2014) was located near a putative TRT locus on CHR 13 identified in the present study. Root weight was also mapped by Brensha et al. (2012), who identified two QTLs for root fresh weight (*qRFW001* and *qRFW002*) and one QTL for root dry weight (*qRDW001*), all of which were located near a putative locus identified for LSLD and PC1 on CHR 8. Interestingly, none of the QTLs identified for root weight by different studies (Zhang et al., 2009; Brensha et al., 2012; Liang et al., 2014) were co-located with each other.

A putative locus for ALD identified on CHR 13 was found near a previously identified QTL for root–shoot weight ratio 2-2 (*qR/S-f-1*) (Wu et al., 2012). A QTL for root morphology 1-2 (*Q_root_Gm03*) on CHR 3 was co-located with the putative locus identified for PC2, and another one (*Q_root_Gm08*) was located close to the LPAR locus identified on CHR 8 (Abdel-Hallem et al., 2011).

Two QTLs for root volume, root volume 1-2 (q*RV1*-*m*-*1*) and root volume 1-3 (q*RV1*-*m*-*2*), were identified to be located near a putative locus for UPAR on CHR 7 (Liang et al., 2014). Root length was previously mapped by different groups (Brensha et al., 2012; Nguyen et al., 2017) in two different RIL populations. A putative locus identified for ALD, PC1, LSLD, and OCS on CHR 14 was located near a QTL (*Qrl-14*) for root length on CHR 14 that was identified by Nguyen et al. (2017), and Brensha et al. (2012) identified two QTLs (*qMRL001* and *qMRL002*) that were located close to a putative locus for PC2 on CHR 6. Additionally, a QTL (*Qhti-14-2*) for root area and two QTLs (*Qrd-14-1* and *Qrd-14-2*) for root diameter and a QTL (*Qcard-14*) for change in root diameter identified (Nguyen et al., 2017) were also found in the same genomic region.

Populations, plant growth conditions, and developmental stages at the time of phenotyping and phenotyping methods differed considerably among the various mapping studies cited above as well as the present study, including growth on paper (phenotyped at V2; Liang et al., 2014), in soil-filled pots (phenotyped 35 days after germination; Zhang et al., 2009), in a modified hydroponic system (phenotyped 13 days after sowing; Nguyen et al., 2017), in pots and later transplanted to the field (phenotyped at R8; Brensha et al., 2012), and under field conditions (phenotyped at R4; Abdel-Hallem et al., 2011). Despite these differences, co-located QTLs among this and other studies indicate genomic regions that may be of particular interest with respect to soybean root system characteristics.

### Putative Candidate Genes and Their Function

A total of 70 candidate genes were identified within ±0.5 Mb of the 67 loci associated with the 14 root traits. Of these candidate genes, 53 and 47% were located within <0.25 and 0.25–0.5 Mb of putative loci, respectively. Functional annotations of candidate genes and follow-up literature search revealed potential root-related functions (Supplementary Table 6).

Among the candidate genes identified for root traits, leucine-rich repeat receptor-protein genes or leucine-rich repeat receptor-like protein kinase genes (*LRR-RPs* or *LRR-RLKs*) were abundant (12 genes) in the vicinity of loci for UPLN, USLD, UPAR, LSLD, LLAA, ALD, PC1, and PC2 (Supplementary Table 6). A potential role of these genes with respect to root traits is consistent with a recent genome-wide analysis of soybean *LRR-RLKs* (Zhou et al., 2016), which suggests that *LRR-RLKs* may function as tissue-specific regulators, including in roots. Interestingly, six *LRR-RLKs* were found in the vicinity of markers for root cone angle in rice (Bettembourg et al., 2017).

Auxin and auxin-related genes are well-known to play important roles in various aspects of root growth and development (Benjamins and Scheres, 2008; Petricka et al., 2012). As such, auxin-related genes located in the vicinity of the identified putative loci were of particular interest and included eight auxin/indole-acetic acid (*AUX/IAA*) family genes, which were found near six putative loci (CHRs 7, 8, 9, 10, 12, and 14) for UPLN, USLD, ULAA, UPAR, LLAA, ALD, and PC2. Aux/IAAs and ARFs are TFs that regulate the cell-specific transcription of auxin response genes for lateral root development (Guilfoyle and Hagen, 2007; Li et al., 2016). Recently, it was documented that miR167 modulated expression of *GmARF8* genes influences not only nodulation but also lateral root numbers in soybean (Wang et al., 2015).

Five serine/threonine-protein kinase-related genes were found close to putative loci on CHRs 1, 3, 5, 7, and 8 identified for TRT, USLD, UPAR, LSLD, and/or PC1. Serine/threonine-protein kinase-related genes are members of the SnRK2 subfamily of the SNF1-related family of protein kinases and include some members that are highly expressed in newly emerged soybean roots (Monks et al., 2001) and *Arabidopsis* lateral roots (Mcloughlin et al., 2012). SnRK2 protein kinases are well-known to be involved in plant responses to abiotic stresses (Kulik et al., 2011), and *GmWNK1*, a member of the WNK kinase family of serine/threonine kinases, has been shown to regulate RSA via an ABA-dependent pathway in soybean (Wang et al., 2010).

MYB-type TFs (MYB TFs) play diverse roles in plant development and stress responses. Four candidate genes were found in the vicinity of four putative loci on CHRs 4, 5, 9, and 10 identified for UPAR, LPAR, LLAA, and NPL. MYB TFs are reported to mediate ABA–auxin cross-talk in drought stress responses and lateral root growth, providing an adaptive strategy under drought (Wang et al., 2017). Additionally, MYB TFs are involved in root elongation (Feng et al., 2004) and root hair patterning (Kang et al., 2009) in *Arabidopsis* and RSA in rice (Dai et al., 2012).

Fasciclin-like arabinogalactan proteins (FLAs) are a subclass of arabinogalactan proteins (AGPs), play a key role in barley root epidermal cell differentiation and root hair development (Marzec et al., 2015), and show higher expression in root tissue during various hormones and stress treatments in *Arabidopsis* (Johnson et al., 2003). Here, four FLA-encoding genes were found near putative loci for UPAR, USLD, LPAR, and ALD on CHRs 2, 6, 8, and 18.

WD40-repeat (WDR) proteins often function as molecular “hubs” mediating supramolecular interactions (Guerriero et al., 2015). In *Medicago truncatula* (Pang et al., 2009) and *Arabidopsis* (Walker et al., 1999), WDR proteins are involved in development of trichomes and root hairs. One WD domain encoding gene on CHR 16 and three WD40 candidate genes were found close to putative loci on CHRs 6, 10, and 17 for UPLN, LLAA, LLAR, UPAR, NPL, ALD, and PC2. Previously, genes encoding proteins containing WD40 repeats were found within 25 kb of putative QTLs that were identified for root angle and total number of tillers in rice (Courtois et al., 2013).

The EF-hand motif is the most common calcium-binding motif found in proteins, and genes encoding EF-hand calcium-binding proteins are preferentially expressed in roots/root tips/root hairs in soybean (Zeng et al., 2017) and in root tips in *Arabidopsis* (Wagner et al., 2015). In this study, three candidate genes near three putative loci on CHRs 5, 9, and 18 associated with OCS, UPAR, USLD, and LSLD were found.

Three lipoxygenase genes were found near two putative loci on CHRs 7 and 10 for LLAA, LLAR, and PC2. Lipoxygenases (linoleate:oxygen oxidoreductase, EC 1.13.11; LOXs) catalyze the conversion of polyunsaturated fatty acids (lipids) into conjugated hydroperoxides. The genome-wide analysis of the lipoxygenase gene family in *Arabidopsis* and rice showed their potential role in lateral root development (Hayashi et al., 2008; Umate, 2011). In soybean, *LOX9* may be involved in root and nodule growth and development, and one of the candidate genes identified here (Glyma.07G034800) was found near a previously studied *LOX* gene (Hayashi et al., 2008).

Other genes found close to putative loci were related to ABC transporter super family, phospholipase D (PLD), BURP domain encoding genes, protein phosphatase 2C (PP2C), ubiquitin C-terminal hydrolases gene (UCHs), glycerophosphodiester phosphodiesterases (GPX-PDE) genes, proline-rich receptor-like protein kinase PER12-related genes, exostosin family protein-related genes, COBRA-like (COB) genes, bHLH protein encoding genes, and TFs, namely, HD-Zip, TPR, AP2/ERF, and WRKY TF family.

This study relied on manual measurements and scoring of root traits, an approach that has been used for a variety of crops (Trachsel et al., 2011; Burridge et al., 2016). With the advent of image-based methods, higher throughput and possibly greater precision of trait measurement due to less time spent evaluating traits and human bias or error (Bucksch et al., 2014; Colombi et al., 2015; Seethepalli et al., 2020) will allow more ambitious studies to expand on the findings reported here. Alleviating the phenotyping bottleneck for roots through the utilization of new technologies like imaging and image analysis for root traits is expected to enhance future genetic and physiological studies. Indeed, physiological studies documenting the influence of roots on crop performance are imperative for the future of crop breeding.

## Conclusions

Substantial heritability for some RSA traits of field-grown soybean reveals promise for breeding, and numerous genetic loci were uncovered for all traits. PCA allowed identification of shared and unique regions associated with multivariate traits. GWAS resulted in the identification of numerous marker–trait associations for each of the RSA trait examined in this study. A total of 67 loci associated with at least one of the 14 root traits were identified. Seventeen loci on 13 CHRs were identified by SNPs associated with more than one root trait. Several gene candidates are proposed for future work to confirm, which will allow studies on contrasting RSAs in the field. A better understanding of the roles and importance of specific root traits with soybean performance in distinct environments and in different soil types, and the identification of associated genetic markers will be critical to strategically target and exploit root characteristics to increase crop yields and ensure cropping system sustainability.

## Data Availability Statement

The 289 genotypes used in this study are part of 19,652 G. max and G. soja accessions genotyped with 52,041 SNPs (http://soybase.org/snps/index.php) of which 31,708 SNPs were used in this study. This information is already available to the public, and the SNP data for our user defined genotypes can be obtained from the following link http://www.soybase.org/snps/download.php. All the data that directly underlie key results and conclusions of this article are attached as Supplementary Material.

## Author Contributions

AD, KH, and FF designed the study. AD and KH performed field experiments and collected phenotype data for RSA traits. AD performed SNP–trait association. AD and LY performed other statistical analyses and wrote the manuscript. FF coordinated and supervised the projects. AD, LY, and FF critically revised the manuscript. All authors read and approved the final manuscript.

## Conflict of Interest

The authors declare that the research was conducted in the absence of any commercial or financial relationships that could be construed as a potential conflict of interest.

## References

[B1] Abdel-Hallem H. Lee G. J. Boerma R. H. (2011). Identification of QTL for increased fibrous roots in soybean. Theor. Appl. Genet. 122, 935–946. 10.1007/s00122-010-1500-9 21165732

[B2] Allmaras R. R. Nelson W. W. Voorhees W. B. (1975). Soybean and corn rooting in southeastern minnesota. II. Root distributions and related water inflow. Soil Sci. Soc. Am. Proc. 39, 771–777. 10.2136/sssaj1975.03615995003900040046x

[B3] Ao J. Fu J. Tian J. Yan X. Liao H. (2010). Genetic variability for root morph-architecture traits and root growth dynamics as related to phosphorus efficiency in soybean. Func. Plant Biol. 37, 304–312. 10.1071/FP09215

[B4] Arya L. M. Blake G. R. Farrell D. A. (1975). A field study of soil water depletion patterns in presence of growing soybean roots: III. Rooting characteristics and root extraction of soil water. Soil Sci. Soc. Am. Proc. 39, 437–444. 10.2136/sssaj1975.03615995003900030023x

[B5] Bacanamwo M. Purcell L. C. (1999). Soybean root morphological and anatomical traits associated with acclimation to flooding. Crop Sci. 39, 143–149. 10.2135/cropsci1999.0011183X003900010023x

[B6] Bass J. D. Swcf A. J. Dabney A. Robinson D. (2015). qvalue: Q-Value Estimation for False Discovery Rate Control. R package version 2.4.2. Available online at: http://github.com/jdstorey/qvalue

[B7] Benjamini Y. Hochberg Y. (1995). Controlling the false discovery rate: a practical and powerful approach to multiple testing. J. R. Stat. Soc. Series B 57, 289–300. 10.1111/j.2517-6161.1995.tb02031.x

[B8] Benjamins R. Scheres B. (2008). Auxin: the looping star in plant development. Annu. Rev. Plant Biol. 59, 443–465. 10.1146/annurev.arplant.58.032806.103805 18444904

[B9] Bettembourg M. Dardou A. Audebert A. Thomas E. Frouin J. Guiderdoni E.. (2017). Genome-wide association mapping for root cone angle in rice. Rice 10:45. 10.1186/s12284-017-0184-z 28971382 PMC5624858

[B10] Bondari K. (2003). Statistical analysis of genotype x environment interaction in agricultural research, in SESUG: The Proceedings of the South East SAS Users Group (St Pete Beach, FL), *Paper SD*15.

[B11] Borst H. L. Thatcher L. E. (1931). Life History and Composition of the Soybean Plant. Research Bulletin 494. Ohio Agricultural Research and Development Center. Columbus, OH, USA: Ohio State Universit 96.

[B12] Bradbury P. J. Zhang Z. Kroon D. E. Casstevens T. M. Ramdoss Y. Buckler E. S. (2007). TASSEL: software for association mapping of complex traits in diverse samples. Bioinformatics 23, 2633–2635. 10.1093/bioinformatics/btm308 17586829

[B13] Brensha W. Kantartzi S. Meksem K. Grier R. Barakat A. Lightfoot D. (2012). Genetic analysis of root and shoot traits in the 'Essex' by 'Forrest' recombinant inbred line (RIL) population of soybean [*Glycine max* (L.) Merr.] J. Plant Genome Sci. 1, 1–9. 10.5147/jpgs.2012.0051

[B14] Brouwer R. (1953). Water absorption by roots of vicia faba at transpiration strengths. Analysis of the uptake and factors determining it. Proc, Koninklijke Nederlandse Akademie Wetenshappen Seriv. 56, 106–115.

[B15] Buckler E. Casstevens T. Bradbury P. Zhang Z. (2009). Analysis by association, Evolution and Linkage (TASSEL) Version 2.1 User Manual. Ithaca, NY: Cornell University.

[B16] Bucksch A. Burridge J. York L. M. Das A. Nord E. Weitz J. S.. (2014). Image-based high-throughput field phenotyping of crop roots. Plant Physiol. 166, 470–486. 10.1104/pp.114.243519 25187526 PMC4213080

[B17] Burch G. L. Smith R. C. G. Mason W. K. (1978). Agronomic and physiological responses of soybean and sorghum crops to water deficits. II. Crop evaporation, soil water depletion and root distribution. Austr. J. Plant Physiol. 5, 169–177. 10.1071/PP9780169

[B18] Burridge J. Jochua C. N. Bucksch A. Lynch J. P. (2016). Legume shovelomics: high—throughput phenotyping of common bean (*Phaseolus vulgaris* L.) and cowpea (vigna unguiculata subsp, unguiculata) root architecture in the field. Field Crops Res. 192, 21–32. 10.1016/j.fcr.2016.04.008

[B19] Chevenet F. Brun C. Banuls A. L. Jacq B. Christen R. (2006). TreeDyn: towards dynamic graphics and annotations for analyses of trees. BMC Bioinformatics 7:439. 10.1186/1471-2105-7-439 17032440 PMC1615880

[B20] Colombi T. Kirchgessner N. Le Marié C. A. York L. M. Lynch J. P. Hund A. (2015). Next generation shovelomics: set up a tent and REST. Plant Soil 388, 1–20. 10.1007/s11104-015-2379-7

[B21] Courtois B. Audebert A. Dardou A. Roques S. Ghneim-Herrera T. Droc G.. (2013). Genome-wide association mapping of root traits in a japonica rice panel. PLoS ONE 8:e78037. 10.1371/journal.pone.0078037 24223758 PMC3818351

[B22] Dai X. Wang Y. Yang A. Zhang W. H. (2012). OsMYB2P-1, an R2R3 MYB transcription factor, is involved in the regulation of phosphate-starvation responses and root architecture in rice. Plant Physiol. 159, 169–183. 10.1104/pp.112.194217 22395576 PMC3375959

[B23] De Dorlodot S. Forster B. Pagès L. Price A. Tuberosa R. Draye X. (2007). Root system architecture: opportunities and constraints for genetic improvement of crops. Trends Plant Sci. 10, 474–481. 10.1016/j.tplants.2007.08.012 17822944

[B24] Den Herder G. Van Isterdael G. Beeckman T. De Smet I. (2010). The roots of a new green revolution. Trends Plant Sci. 15, 600–607. 10.1016/j.tplants.2010.08.009 20851036

[B25] Dhanapal A. P. Ray J. D. Singh S. K. Hoyos-Villegas V. Smith J. R. Purcell L. C. (2015a). Genome-wide association study (GWAS) of carbon isotope ratio (δ13C) in diverse soybean [*Glycine max* (L.) Merr.] genotypes. Theor. Appl. Genet. 128, 73–91. 10.1007/s00122-014-2413-9 25367378

[B26] Dhanapal A. P. Ray J. D. Singh S. K. Hoyos-Villegas V. Smith J. R. Purcell L. C.. (2015b). Genome-wide association analysis of diverse soybean genotypes reveals novel markers for Nitrogen derived from atmosphere (Ndfa), nitrogen concentration ([N]) and C/N ratio. Plant Genome 8:86. 10.3835/plantgenome2014.11.0086 33228264

[B27] Dhanapal A. P. Ray J. D. Singh S. K. Hoyos-Villegas V. Smith J. R. Purcell L. C. (2015c). Association mapping of total carotenoids in diverse soybean genotypes based on leaf extracts and high-throughput canopy spectral reflectance measurements. PLoS ONE 10:e0137213 10.1371/journal.pone.0137213 26368323 PMC4569184

[B28] Endelman J. B. Jannink J. L. (2012). Shrinkage estimation of the realized relationship matrix. G3 2, 1405–1413. 10.1534/g3.112.004259 23173092 PMC3484671

[B29] Feng C. Andressson E. Maslak A. Mock H. P. Mattsson O. Mundy L. (2004). Arabidopsis MYB68 in development and responses to environmental cues. Plant Sci. 167, 1099–1107. 10.1016/j.plantsci.2004.06.014

[B30] Fenta B. Beebe S. Kunert K. Burridge J. Barlow K. Lynch J. (2014). Field phenotyping of soybean roots for drought stress tolerance. Agronomy 4, 418–435. 10.3390/agronomy4030418

[B31] Fenta B. A. Schlüter U. Garcia B. M. Duplessis M. Foyer C. H. Kunert K. J. (2011). Identification and application of phenotypic and molecular markers for abiotic stress tolerance in soybean, in Soybean—Genetics and Novel Techniques for Yield Enhancement, D. Krezhova (Shanghai, China: InTech), 181–200.

[B32] Fitter A. H. Stickland T. R. Harvey M. L. Wilson G. W. (1991). Architectural analysis of plant root systems. 1. Architectural correlates of exploitation efficiency. N. Phytol. 118, 375–382. 10.1111/j.1469-8137.1991.tb00018.x

[B33] Galesloot T. E. Van Steen K. Kiemeney L. A. Janss L. L. Vermeulen S. H. (2014). A comparison of multivariate genome-wide association methods. PLoS ONE 9:e95923. 10.1371/journal.pone.0095923 24763738 PMC3999149

[B34] Grant D. Nelson R. T. Cannon S. C. (2010). SoyBase, the USDA-ARS Genetics and Genomics Database. [WWW document] URL Available online at: http://soybase.org 10.1093/nar/gkp798 PMC2808871 20008513

[B35] Gruber B. D. Giehl R. F. Friedel S. Von Wirén N. (2013). Plasticity of the arabidopsis root system under nutrient deficiencies. Plant Physiol. 163, 161–179. 10.1104/pp.113.218453 23852440 PMC3762638

[B36] Guerriero G. Hausman J. F. Ezcurra I. (2015). WD40-repeat proteins in plant cell wall formation: current evidence and research prospects. Front. Plant Sci. 6:1112. 10.3389/fpls.2015.01112 26734023 PMC4686805

[B37] Guilfoyle T. J. Hagen G. (2007). Auxin response factors. Curr. Opin. Plant Biol. 5, 453–460. 10.1016/j.pbi.2007.08.014 17900969

[B38] Ha C. V. Le D. T. Nishiyama R. Watanabe Y. Tran U. T. Dong N. V.. (2013). Characterization of the newly developed soybean cultivar DT2008 in relation to the model variety W82 reveals a new genetic resource for comparative and functional genomics for improved drought tolerance. Biomed Res. Int. 2013:759657. 10.1155/2013/759657 23509774 PMC3591244

[B39] Hayashi S. Gresshoff P. M. Kinkema M. (2008). Molecular analysis of lipoxygenases associated with nodule development in soybean. Mol. Plant Microbe Interact. 21, 843–853. 10.1094/MPMI-21-6-0843 18624647

[B40] He J. Jin Y. Du Y. L. Wang T. Turner N. C. Yang R. P.. (2017). Genotypic variation in yield, yield components, root morphology and architecture, in soybean in relation to water and phosphorus supply. Front. Plant Sci. 8:1499. 10.3389/fpls.2017.01499 28912792 PMC5583600

[B41] Herritt M. Dhanapal A. P. Fritschi F. B. (2016). Identification of genomic loci associated with the photochemical reflectance index by genome-wide association study in soybean. Plant Genome 9:72. 10.3835/plantgenome2015.08.0072 27898827

[B42] Ho M. D. Mccannon B. C. Lynch J. P. (2004). Optimization modeling of plant root architecture for water and phosphorus acquisition. J. Theor. Biol. 226, 331–340. 10.1016/j.jtbi.2003.09.011 14643647

[B43] Ho M. D. Rosas J. C. Brown K. M. Lynch J. P. (2005). Root architectural tradeoffs for water and phosphorus acquisition. Func. Plant Biol. 32, 737–748. 10.1071/FP05043 32689171

[B44] Houx J. H. III Wiebold W. J. Fritschi F. B. (2011). Long-term tillage and crop rotation determines the mineral nutrient distributions of some elements in a vertic epiaqualf. Soil Tillage Res. 112, 27–35. 10.1016/j.still.2010.11.003

[B45] Jobbagy E. G. Jackson R. B. (2001). The distribution of soil nutrients with depth: global patterns and the imprint of plants. Biogeochemistry 53, 51–77. 10.1023/A:1010760720215

[B46] Johnson K. L. Jones B. J. Bacic A. Schultz C. J. (2003). The fasciclin-like arabinogalactan proteins of arabidopsis. A multigene family of putative cell adhesion molecules. Plant Physiol. 133, 1911–1925. 10.1104/pp.103.031237 14645732 PMC300743

[B47] Kang Y. H. Kirik V. Hulskamp M. Nam K. H. Hagely K. Lee M. M.. (2009). The MYB23 gene provides a positive feedback loop for cell fate specification in the arabidopsis root epidermis. Plant Cell 21, 1080–1094. 10.1105/tpc.108.063180 19395683 PMC2685616

[B48] Kaspar T. C. Taylor H. M. Shibles R. M. (1984). Taproot-elongation rates of soybean cultivars in the glasshouse and their relation to field rooting depth. Crop Sci. 24, 916–920. 10.2135/cropsci1984.0011183X002400050021x

[B49] Kulik A. Wawer I. Krzywińska E. Bucholc M. Dobrowolska G. (2011). SnRK2 protein kinases–key regulators of plant response to abiotic stresses. OMICS 15, 859–872. 10.1089/omi.2011.0091 22136638 PMC3241737

[B50] Lersten N. R. Carlson J. B. (2004). Vegetative morphology, in Soybeans: Improvement, Production and Uses, 3 ed eds Boerma H. R. Specht J. E. (Madison, WI: American Society of Agronomy, Crop Science Society of America, Soil Science Society of America), 15–57.

[B51] Li S. B. Xie Z. Z. Hu C. G. Zhang J. Z. (2016). A review of auxin response factors (ARFs) in plants. Front. Plant Sci. 7:47. 10.3389/fpls.2016.00047 26870066 PMC4737911

[B52] Liang H. Yu Y. Yang H. Xu L. Dong W. Du H.. (2014). Inheritance and QTL mapping of related root traits in soybean at the seedling stage. Theor. Appl. Genet. 127, 2127–2137. 10.1007/s00122-014-2366-z 25145446

[B53] Liang Q. Cheng X. Mei M. Yan X. Liao H. (2010). QTL analysis of root traits as related to phosphorus efficiency in soybean. Ann. Bot. 106, 223–234. 10.1093/aob/mcq097 20472699 PMC2889805

[B54] Liao H. Yan X. Rubio G. Beebe S. E. S. Blair M. M. W. Lynch J. P. (2004). Genetic mapping of basal root gravitropism and phosphorus acquisition efficiency in common bean. Func. Plant Biol. 31, 959–970. 10.1071/FP03255 32688964

[B55] Lipka A. E. Tian F. Wang Q. Peiffer J. Li M. Bradbury P. J.. (2012). GAPIT: genome association and prediction integrated tool. Bioinformatics 28, 2397–2399. 10.1093/bioinformatics/bts444 22796960

[B56] Lynch J. P. (1995). Root architecture and plant productivity. Plant Physiol. 109, 7–13. 10.1104/pp.109.1.7 12228579 PMC157559

[B57] Lynch J. P. (2013). Steep, cheap and deep: an ideotype to optimize water and N acquisition by maize root systems. Ann. Bot. 112, 347–357. 10.1093/aob/mcs293 23328767 PMC3698384

[B58] Malamy J. E. (2005). Intrinsic and environmental response pathways that regulate root system architecture. Plant Cell Environ. 28, 67–77 10.1111/j.1365-3040.2005.01306.x 16021787

[B59] Malamy J. E. Benfey P. N. (1997). Organization and cell differentiation in lateral roots of arabidopsis thaliana. Development 124, 33–44. 9006065 10.1242/dev.124.1.33

[B60] Manavalan L. P. Guttikonda S. K. Tran L. S. Nguyen H. T. (2009). Physiological and molecular approaches to improve drought resistance in soybean. Plant Cell Physiol. 50, 1260–1276. 10.1093/pcp/pcp082 19546148

[B61] Manavalan L. P. Prince S. J. Musket T. A. Chaky J. Deshmukh R. Vuong T. D.. (2015). Identification of novel QTL governing root architectural traits in an interspecific soybean population. PLoS ONE 10:e0120490. 10.1371/journal.pone.0120490 25756528 PMC4355624

[B62] Marzec M. Szarejko I. Melzer M. (2015). Arabinogalactan proteins are involved in root hair development in barley. J. Exp. Bot. 66, 1245–1257. 10.1093/jxb/eru475 25465033 PMC4339589

[B63] Matsuo N. Takahashi M. Fukami K. Tsuchiya S. Tasaka K. (2013). Root growth of two soybean cultivars grown under different groundwater level conditions. Plant Prod. Sci. 16, 374–382. 10.1626/pps.16.374

[B64] Mcloughlin F. Galvan-Ampudia C. S. Julkowska M. M. Caarls L. Van Der Does D. Laurière C.. (2012). The Snf1-related protein kinases SnRK2.4 and SnRK2.10 are involved in maintenance of root system architecture during salt stress. Plant J. 72, 436–449. 10.1111/j.1365-313X.2012.05089.x 22738204 PMC3533798

[B65] Monks D. Aghoram K. Courtney P. Dewald D. Dewey R. E. (2001). Hyperosmotic stress induces the rapid phosphorylation of a soybean phosphatidylinositol transfer protein homolog through activation of the protein kinases SPK1 and SPK2. Plant Cell 5, 1205–1219. 10.1105/tpc.13.5.1205 PMC135558 11340192

[B66] Morales A. M. O'rourke J. A. Van De Mortel M. Scheider K. T. Bancroft T. J. Borém A.. (2013). Transcriptome analyses and virus induced gene silencing identify genes in the Rpp4-mediated Asian soybean rust resistance pathway. Func. Plant Biol. 40, 1029–1047. 10.1071/FP12296 32481171

[B67] Nguyen L. Takahashi R. Githiri S. Rodriguez T. Tsutsumi N. Kajihara S.. (2017). Mapping quantitative trait loci for root development under hypoxia conditions in soybean (*Glycine max* L. Merr.) Theor. Appl. Genet. 130, 743–755. 10.1007/s00122-016-2847-3 28097398

[B68] Osmont K. S. Sibout R. Hardtke C. S. (2007). Hidden branches: developments in root system architecture. Annu. Rev. Plant Biol. 58, 93–113. 10.1146/annurev.arplant.58.032806.104006 17177637

[B69] Pacheco-Villalobos D. Hardtke C. S. (2012). Natural genetic variation of root system architecture from arabidopsis to brachypodium: towards adaptive value. Philos. Trans. R. Soc. B Biol. Sci. 367, 1552–1558. 10.1098/rstb.2011.0237 22527398 PMC3321687

[B70] Pang Y. Wenger J. P. Saathoff K. Peel G. J. Wen J. Huhman D. (2009). A WD40 repeat protein from medicago truncatula is necessary for tissue-specific anthocyanin and proanthocyanidin biosynthesis but not for trichome development. Plant Physiol. 151, 1114–1129. 10.1104/pp.109.144022 19710231 PMC2773055

[B71] Péret B. De Rybel B. Casimiro I. Benková E. Swarup R. Laplaze L.. (2009). Arabidopsis lateral root development: an emerging story. Trends Plant Sci. 14, 399–408. 10.1016/j.tplants.2009.05.002 19559642

[B72] Petricka J. J. Winter C. M. Benfey P. N. (2012). Control of arabidopsis root development. Annu. Rev. Res. Plant Biol. 63, 563–590. 10.1146/annurev-arplant-042811-105501 22404466 PMC3646660

[B73] Piepho H. P. (1994). Best linear unbiased prediction (BLUP) for regional yield trials: a comparison to additive main effects and multiplicative interaction (AMMI) analysis. Theor. Appl. Genet. 89, 647–654. 10.1007/BF00222462 24177943

[B74] Piepho H. P. Möhring J. (2007). Computing heritability and selection response from unbalanced plant breeding trials. Genetics 177, 1881–1888. 10.1534/genetics.107.074229 18039886 PMC2147938

[B75] Pritchard J. Stephens M. Donnelly P. (2000). Inference of population structure using multilocus genotype data. Genetics 155, 945–959. 10835412 10.1093/genetics/155.2.945 PMC1461096

[B76] Read D. J. Bartlett E. M. (1972). The physiology of drought resistance in the soy-bean plant (Glycine max) the relationship between drought resistance and growth. J. Appl. Ecol. 9, 487–499. 10.2307/2402447

[B77] Rich S. M. Watt M. (2013). Soil conditions and cereal root system architecture: review and considerations for linking darwin and weaver. J. Exp. Bot. 64, 1193–1208. 10.1093/jxb/ert043 23505309

[B78] Sas-Institute-Inc (2004). SAS/STAT User's Guide Version 9.2 Cary, NC: SAS-Institute-Inc.

[B79] Seethepalli A. Guo H. Liu X. Griffiths M. Almtarfi H. Li Z.. (2020). RhizoVision crown: an integrated hardware and software platform for root crown phenotyping. Plant Phenomics 2020:3074916. 10.34133/2020/3074916 33313547 PMC7706346

[B80] Silvius J. E. Johnson R. R. Peters D. B. (1977). Effect of water stress on carbon assimilation and distribution in soybean plants at different stages of development. Crop Sci. 17, 713–716. 10.2135/cropsci1977.0011183X001700050010x

[B81] Smith S. De Smet I. (2012). Root system architecture: insights from arabidopsis and cereal crops. Philos. Trans. R. Soc. B Biol. Sci. 367, 1441–1452. 10.1098/rstb.2011.0234 22527386 PMC3321685

[B82] Song Q. Hyten D. L. Jia G. Quigley C. V. Fickus E. W. Nelson R. L.. (2013). Development and evaluation of SoySNP50K, a high-density genotyping array for soybean. PLoS ONE 8:e54985. 10.1371/journal.pone.0054985 23372807 PMC3555945

[B83] Storey J. D. Tibshirani R. (2003). Statistical significance for genomewide studies. Proc. Natl. Acad. Sci. U.S.A. 100, 9440–9445. 10.1073/pnas.1530509100 12883005 PMC170937

[B84] Trachsel S. Kaeppler S. M. Brown K. M. Lynch J. P. (2011). Shovelomics: high throughput phenotyping of maize (*Zea mays* L.) root architecture in the field. Plant Soil 341, 75–87. 10.1007/s11104-010-0623-8

[B85] Umate P. (2011). Genome-wide analysis of lipoxygenase gene family in arabidopsis and rice. Plant Signal. Behav. 6, 335–338. 10.4161/psb.6.3.13546 21336026 PMC3142411

[B86] Wagner S. Behera S. De Bortoli S. Logan D. C. Fuchs P. Carraretto L.. (2015). The EF-hand Ca2+ binding protein MICU choreographs mitochondrial Ca2+ dynamics in arabidopsis. Plant Cell 27, 3190–3212. 10.1105/tpc.15.00509 26530087 PMC4682298

[B87] Waines J. G. Ehdaie B. (2007). Domestication and crop physiology: roots of green-revolution wheat. Ann. Bot. 100, 991–998. 10.1093/aob/mcm180 17940075 PMC2759207

[B88] Waisel Y. Eshel A. Kafk Afi U. (2002). Plant Roots the Hidden Half. 3rd Edn New York: Marcel Dekker 10.1201/9780203909423

[B89] Walker A. R. Davison P. A. Bolognesi-Winfield A. C. James C. M. Srinivasan N. Tl B.. (1999). The TRANSPARENT TESTA GLABRA1 locus, which regulates trichome differentiation and anthocyanin biosynthesis in arabidopsis, encodes a WD40 repeat protein. Plant Cell 11, 1337–1349. 10.1105/tpc.11.7.1337 10402433 PMC144274

[B90] Wang L. Liao H. Yan X. Zhuang B. Dong Y. (2004). Genetic variability for root hair traits as related to phosphorus status in soybean. Plant Soil 261, 77–84. 10.1023/B:PLSO.0000035552.94249.6a

[B91] Wang N. Zhang W. Qin M. Li S. Qiao M. Liu Z. (2017). Drought tolerance conferred in soybean (*Glycine max*. L) by GmMYB84, a novel R2R3-MYB transcription factor. Plant Cell Physiol. 58, 1764–1776. 10.1093/pcp/pcx111 29016915

[B92] Wang Y. Li K. Chen L. Zou Y. Liu H. Tian Y.. (2015). MicroRNA167-directed regulation of the auxin response factors GmARF8a and GmARF8b is required for soybean nodulation and lateral root development. Plant Physiol. 168, 984–999. 10.1104/pp.15.00265 25941314 PMC4741323

[B93] Wang Y. Suo H. Zheng Y. Liu K. Zhuang C. Kahle K. T.. (2010). The soybean root-specific protein kinase GmWNK1 regulates stress-responsive ABA signaling on the root system architecture. Plant J. 2, 230–242. 10.1111/j.1365-313X.2010.04320.x 20735771

[B94] Wu J. J. Xu P. F. Liu L. J. Zhang S. Wang J. S. Lin W. G. (2012). Mapping QTLs for phosphorus-deficiency tolerance in soybean at seedling stage, in 2012 International Conference on Biomedical Engineering and Biotechnology (Macao: IEEE), 370–378. 10.1109/iCBEB.2012.269

[B95] York L. M. (2019). Functional phenomics: an emerging field integrating high-throughput phenotyping, physiology, and bioinformatics. J. Exp. Bot. 70, 379–386. 10.1093/jxb/ery379 30380107

[B96] York L. M. Lynch J. P. (2015). Intensive field phenotyping of maize (*Zea mays* L.) root crowns identifies phenes and phene integration associated with plant growth and nitrogen acquisition. J. Exp. Bot. 66, 5493–5505. 10.1093/jxb/erv241 26041317 PMC4585417

[B97] York L. M. Nord E. A. Lynch J. P. (2013). Integration of root phenes for soil resource acquisition. Front. Plant Sci. 4:355. 10.3389/fpls.2013.00355 24062755 PMC3771073

[B98] York L. M. (2018). Phenotyping crop root crowns: general guidance and specific protocols for maize, wheat, and soybean, in Root Development: Methods and Protocols, eds Ristova D. Barbez E. (New York, NY: Springer), 23–32. 10.1007/978-1-4939-7747-5_2 29525946

[B99] Zeng H. Zhang Y. Zhang X. Pi E. Zhu Y. (2017). Analysis of EF-hand proteins in soybean genome suggests their potential roles in environmental and nutritional stress signaling. Front. Plant Sci. 8:877. 10.3389/fpls.2017.00877 28596783 PMC5443154

[B100] Zhang D. Cheng H. Geng L. Kan G. Cui S. Meng Q. (2009). Detection of quantitative trait loci for phosphorus deficiency tolerance at soybean seedling stage. Euphytica 167, 313–322. 10.1007/s10681-009-9880-0

[B101] Zhang Z. Ersoz E. Lai C. Q. Todhunter R. J. Tiwari H. K. Gore M. A.. (2010). Mixed linear model approach adapted for genome-wide association studies. Nat. Genet. 42, 355–360. 10.1038/ng.546 20208535 PMC2931336

[B102] Zhao J. Fu J. Liao H. He Y. Nian H. Hu Y. (2004). Characterization of root architecture in an applied core collection for Phosphorus efficiency of soybean germplasm. Chin. Sci. Bull. 49, 1611–1620. 10.1007/BF03184131

[B103] Zhou F. Guo Y. Qiu L. J. (2016). Genome-wide identification and evolutionary analysis of leucine-rich repeat receptor-like protein kinase genes in soybean. BMC Plant Biol. 16:58. 10.1186/s12870-016-0744-1 26935840 PMC4776374

[B104] Zhu J. Ingram P. A. Benfey P. N. Elich T. (2011). From lab to field, new approaches to phenotyping root system architecture. Curr. Opin. Plant Biol. 14, 310–317. 10.1016/j.pbi.2011.03.020 21530367

