# Supplementary figures and images for: Genome-Wide Association Study of Topsoil Root System Architecture in Field-Grown Soybean [Glycine max (L.) Merr.]
Source: Front Plant Sci. 2021 Feb 10;11:590179. doi: 10.3389/fpls.2020.590179 (PMC7902768; doi:10.3389/fpls.2020.590179)

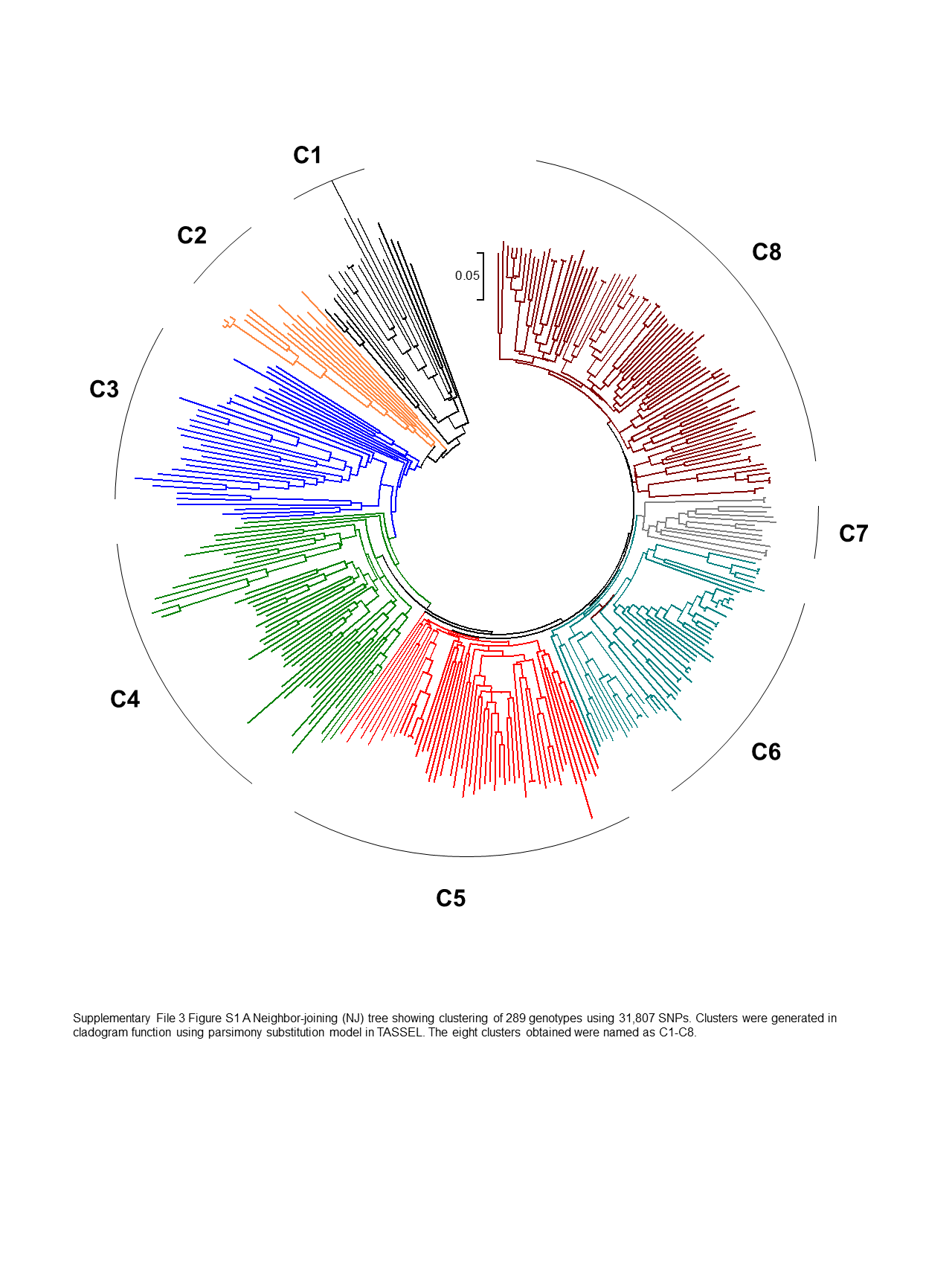

Supplement: Supplementary file 1 [file Image_1.TIF]

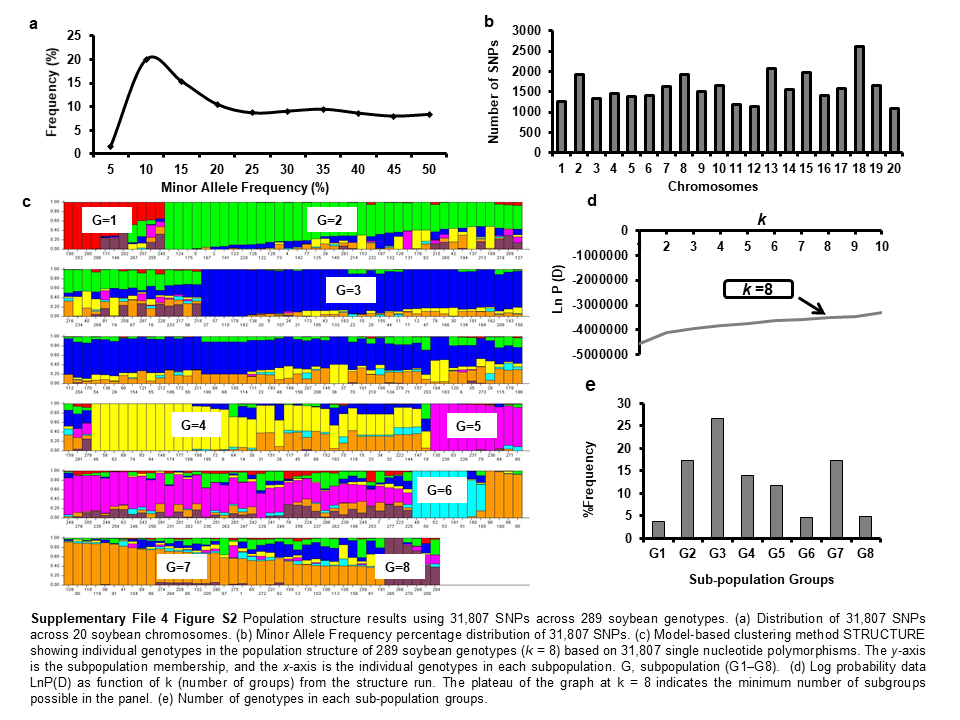

Supplement: Supplementary file 2 [file Image_2.TIF]

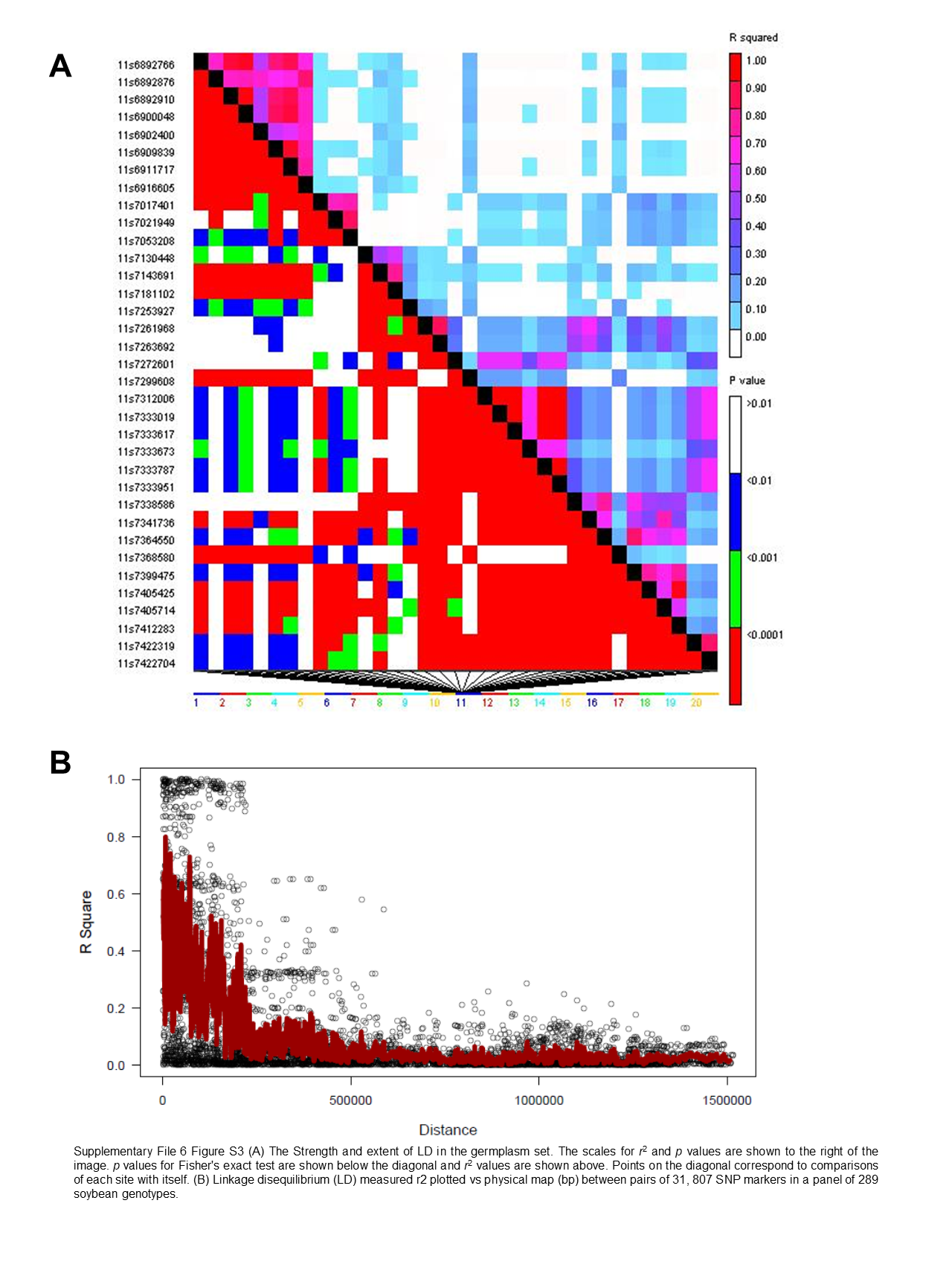

Supplement: Supplementary file 3 [file Image_3.TIF]

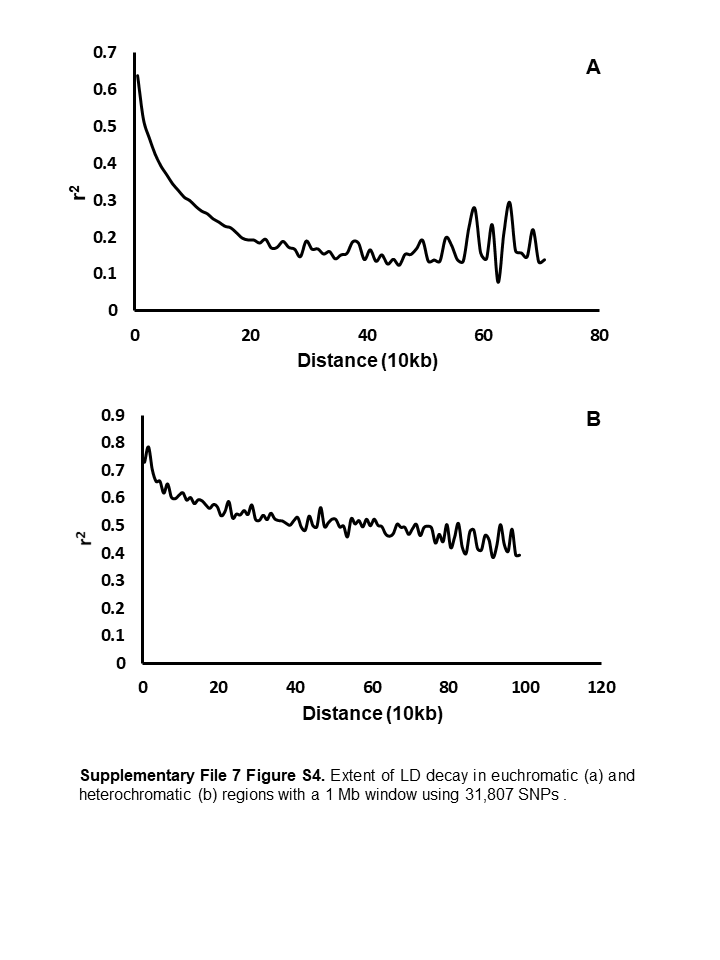

Supplement: Supplementary file 4 [file Image_4.TIF]

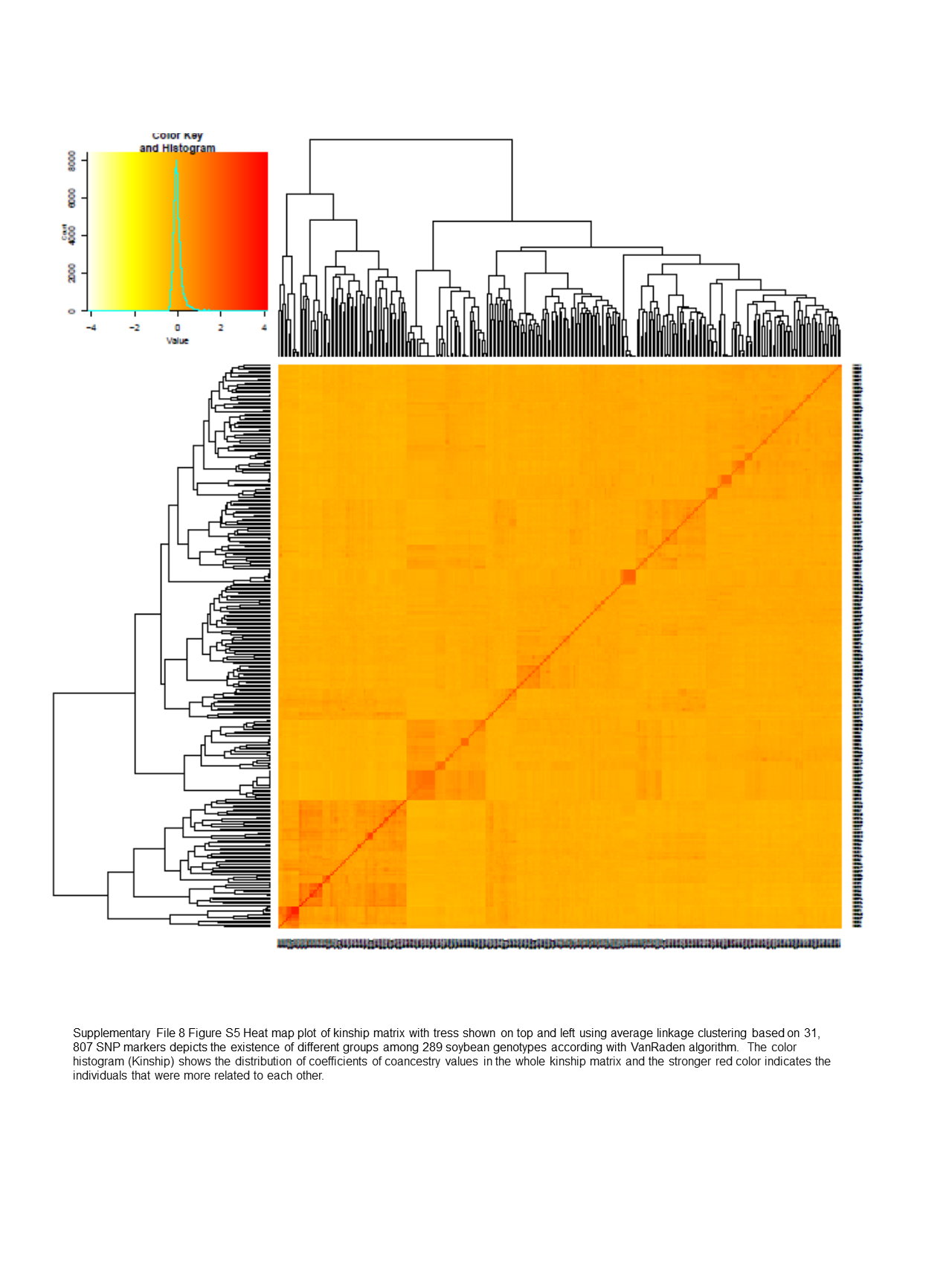

Supplement: Supplementary file 5 [file Image_5.TIF]

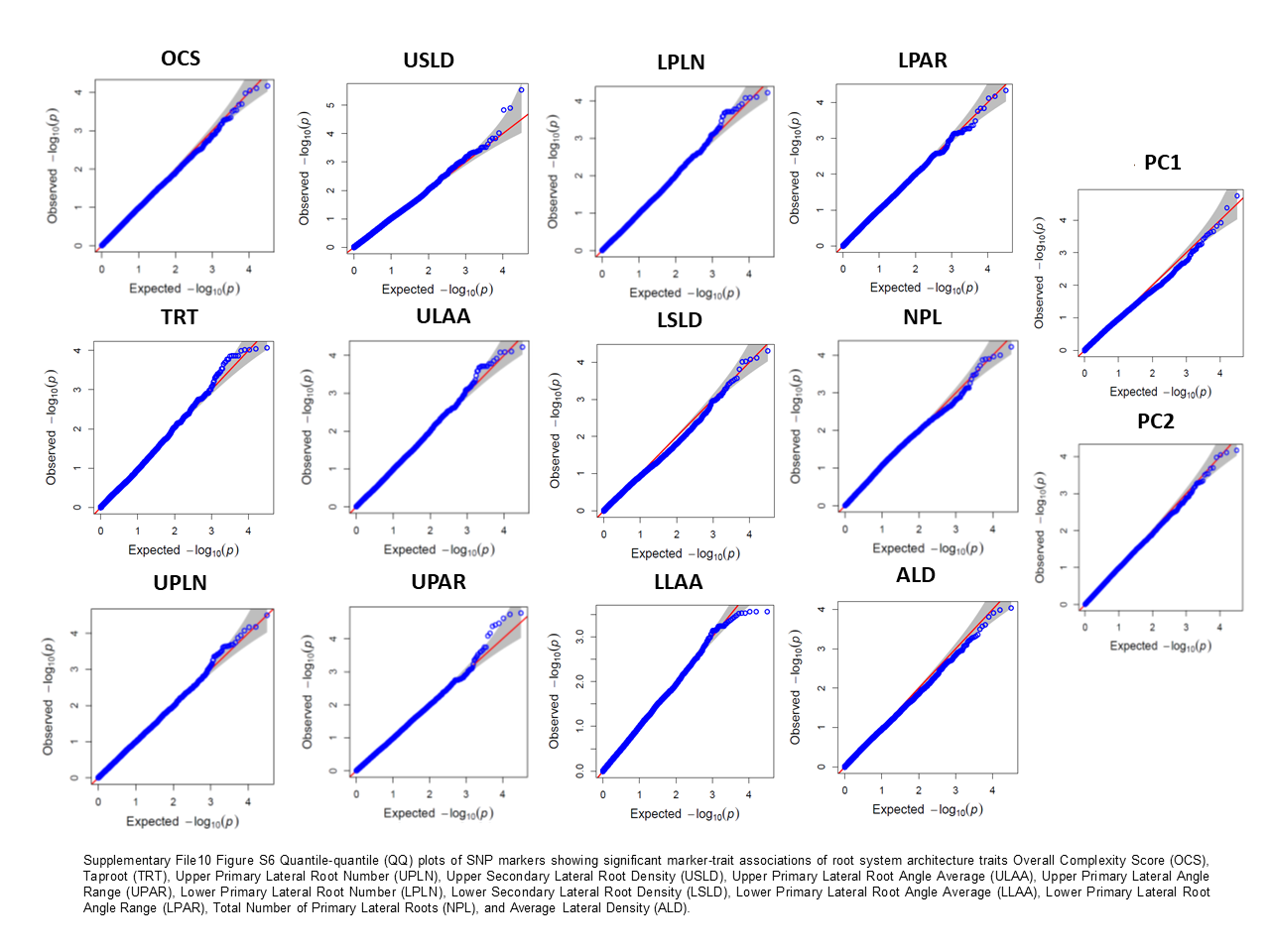

Supplement: Supplementary file 6 [file Image_6.TIF]

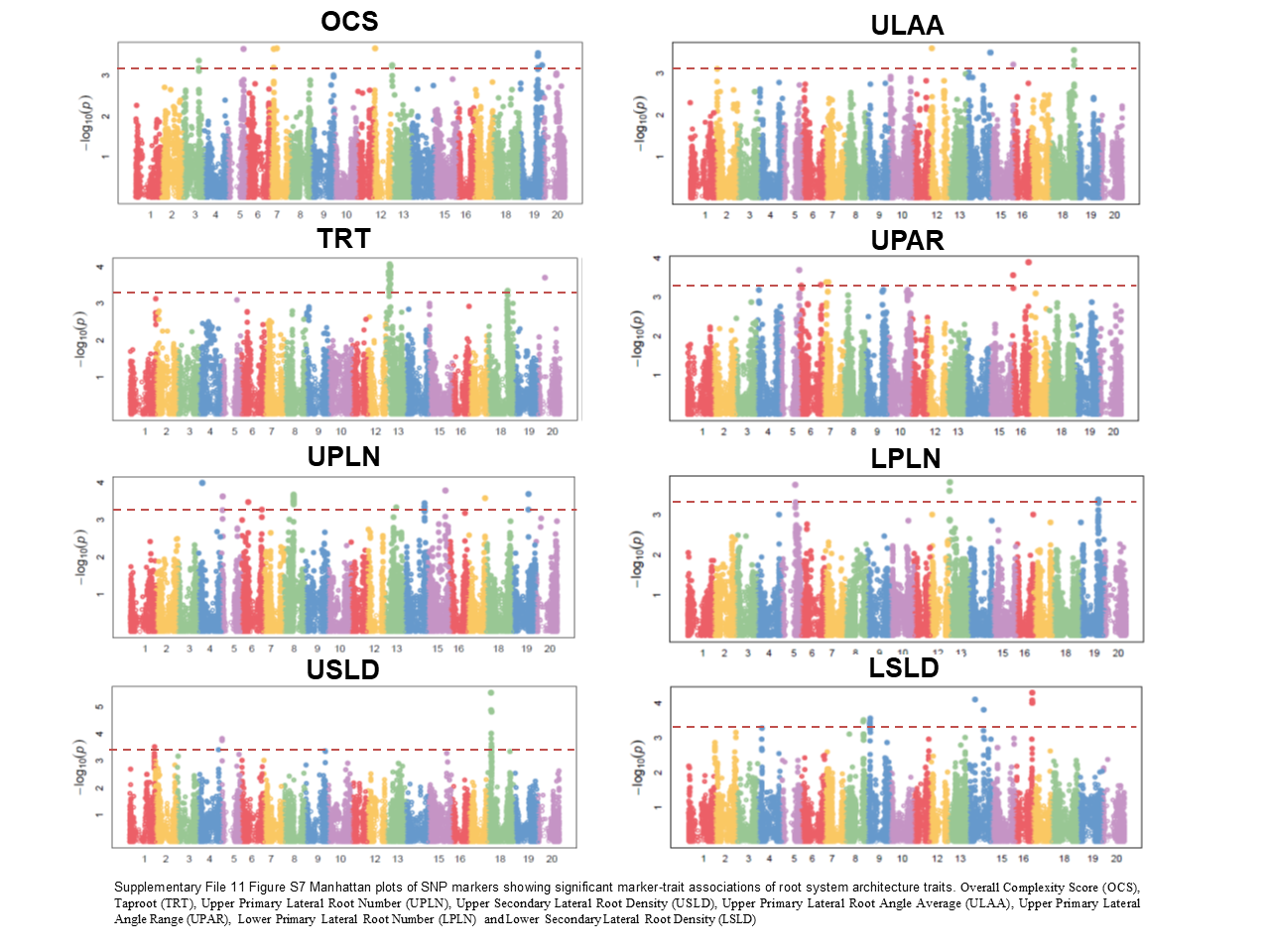

Supplement: Supplementary file 7 [file Image_7.TIF]

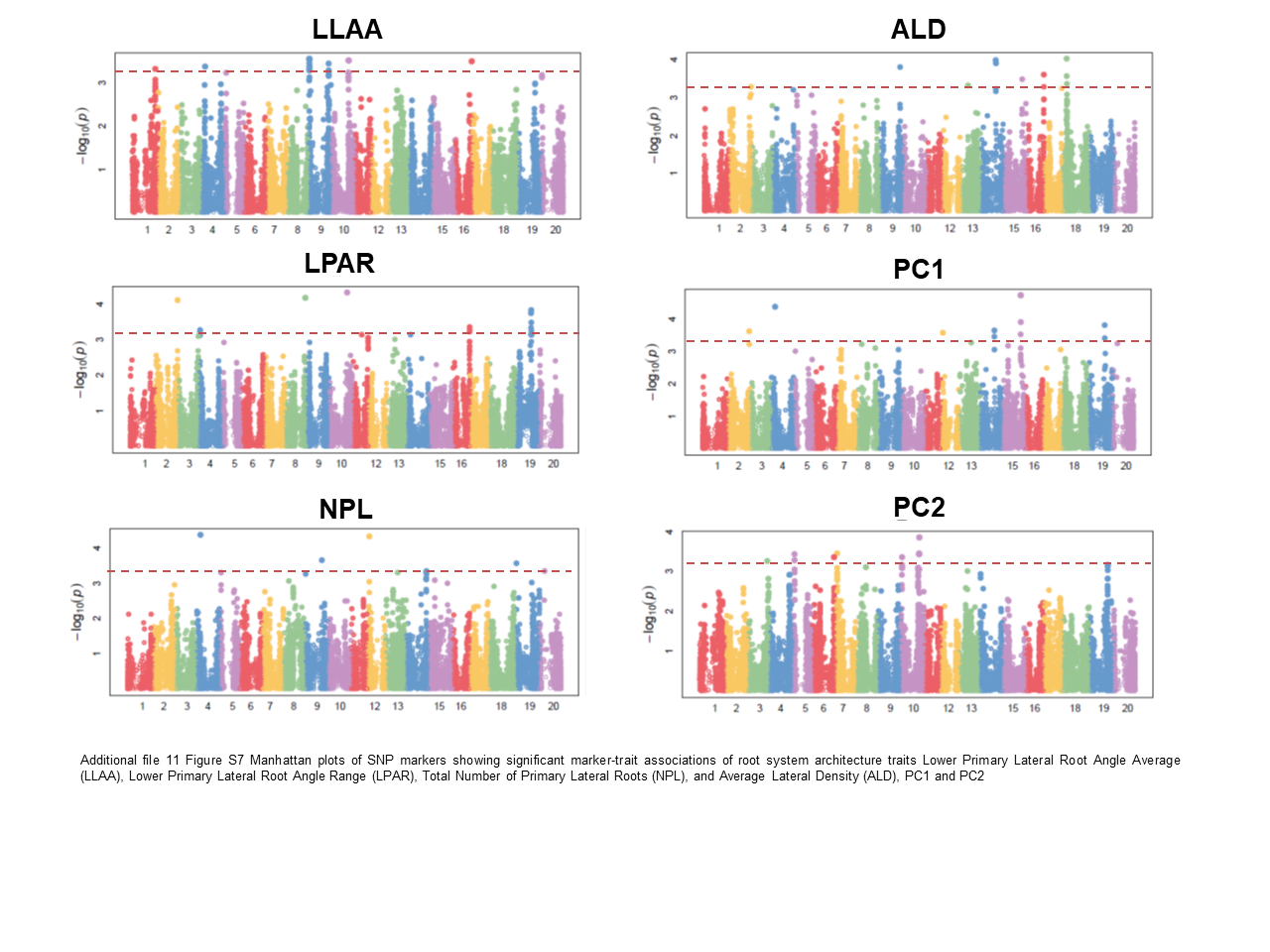

Supplement: Supplementary file 8 [file Image_8.TIF]
