# Supplementary material for: Genome-Wide Association Study of Topsoil Root System Architecture in Field-Grown Soybean [Glycine max (L.) Merr.]
Source: Front Plant Sci. 2021 Feb 10;11:590179. doi: 10.3389/fpls.2020.590179 (PMC7902768; doi:10.3389/fpls.2020.590179)
Supplement: Supplementary file 10 [file Table_2.DOCX]

**Supplementary File 2 Table S2** Analysis of variance of root system architecture traits of 289 soybean genotypes evaluated at Rollins Bottom in 2012 and 2013 and at Rhodes in 2013. using raw phenotypic data and BLUP. Overall Complexity Score (OCS), Taproot (TRT), Upper Primary Lateral Root Number (UPLN), Upper Secondary Lateral Root Density (USLD), Upper Primary Lateral Root Angle Average (ULAA), Upper Primary Lateral Angle Range (UPAR), Lower Primary Lateral Root Number (LPLN), Lower Secondary Lateral Root Density (LSLD), Lower Primary Lateral Root Angle Average (LLAA), Lower Primary Lateral Root Angle Range (LPAR), Total Number of Primary Lateral Roots (NPL), and Average Lateral Density (ALD).

| **Name of Trait** | **Source of Variation** | **Raw Phenotypic Data** | | | **BLUP Data** | | |
| --- | --- | --- | --- | --- | --- | --- | --- |
|  |  | ***df*** | ***F* value** | ***P* value** | ***df*** | ***F* value** | ***P* value** |
| **OCS** | Environment | 2 | 67.73 | <0.001 | 2 | 35.31 | <0.001 |
|  | Genotype | 288 | 1.77 | <0.001 | 288 | 1.63 | <0.001 |
|  | Environment x Genotype | 564 | 1.31 | <0.001 | 564 | 1.22 | <0.001 |
| **TRT** | Environment | 2 | 47.83 | <0.001 | 2 | 33.24 | <0.001 |
|  | Genotype | 288 | 1.11 | <0.001 | 288 | 0.97 | <0.001 |
|  | Environment x Genotype | 564 | 1.04 | 0.2896 | 564 | 0.88 | <0.001 |
| **UPLN** | Environment | 2 | 500.24 | <0.001 | 2 | 430.27 | <0.001 |
|  | Genotype | 288 | 1.64 | <0.001 | 288 | 1.41 | <0.001 |
|  | Environment x Genotype | 564 | 1.46 | <0.001 | 564 | 1.28 | <0.001 |
| **USLD** | Environment | 2 | 147.00 | <0.001 | 2 | 132.02 | <0.001 |
|  | Genotype | 288 | 1.04 | <0.001 | 288 | 0.83 | <0.001 |
|  | Environment x Genotype | 560 | 1.09 | 0.1134 | 560 | 0.92 | <0.001 |
| **ULAA** | Environment | 2 | 325.25 | <0.001 | 2 | 263.42 | <0.001 |
|  | Genotype | 288 | 1.02 | <0.001 | 288 | 0.92 | <0.001 |
|  | Environment x Genotype | 560 | 1.12 | 0.0480 | 560 | 0.79 | <0.001 |
| **UPAR** | Environment | 2 | 96.64 | <0.001 | 2 | 77.47 | <0.001 |
|  | Genotype | 288 | 1.33 | <0.001 | 288 | 1.04 | <0.001 |
|  | Environment x Genotype | 560 | 1.08 | 0.1428 | 560 | 0.94 | <0.001 |
| **LPLN** | Environment | 2 | 56.78 | <0.001 | 2 | 45.32 | <0.001 |
|  | Genotype | 288 | 1.55 | <0.001 | 288 | 1.31 | <0.001 |
|  | Environment x Genotype | 564 | 1.37 | <0.001 | 564 | 1.24 | <0.001 |
| **LSLD** | Environment | 2 | 56.78 | <0.001 | 2 | 43.65 | <0.001 |
|  | Genotype | 288 | 1.55 | <0.001 | 288 | 1.32 | <0.001 |
|  | Environment x Genotype | 564 | 1.37 | 0.1456 | 564 | 1.26 | <0.001 |
| **LLAA** | Environment | 2 | 476.43 | <0.001 | 2 | 374.32 | <0.001 |
|  | Genotype | 288 | 1.37 | <0.001 | 288 | 1.26 | <0.001 |
|  | Environment x Genotype | 564 | 1.13 | 0.0387 | 564 | 0.95 | <0.001 |
| **LPAR** | Environment | 2 | 219.00 | <0.001 | 2 | 187.62 | <0.001 |
|  | Genotype | 288 | 1.26 | <0.001 | 288 | 1.13 | <0.001 |
|  | Environment x Genotype | 564 | 1.09 | 0.1031 | 564 | 0.86 | <0.001 |
| **NPL** | Environment | 2 | 374.23 | <0.001 | 2 | 312.47 | <0.001 |
|  | Genotype | 288 | 1.71 | <0.001 | 288 | 1.41 | <0.001 |
|  | Environment x Genotype | 564 | 1.58 | <0.001 | 564 | 1.38 | <0.001 |
| **ALD** | Environment | 2 | 144.63 | <0.001 | 2 | 133.41 | <0.001 |
|  | Genotype | 288 | 1.14 | <0.001 | 288 | 1.02 | <0.001 |
|  | Environment x Genotype | 564 | 1.11 | 0.0627 | 564 | 1.01 | <0.001 |
