# Supplementary material for: Genome-Wide Association Study of Topsoil Root System Architecture in Field-Grown Soybean [Glycine max (L.) Merr.]
Source: Front Plant Sci. 2021 Feb 10;11:590179. doi: 10.3389/fpls.2020.590179 (PMC7902768; doi:10.3389/fpls.2020.590179)
Supplement: Supplementary file 11 [file Table_3.DOCX]

**Supplementary File 5 Table S3.** The country of origin distribution of the 289 genotypes arranged by eight subpopulation and average distances (expected heterozygosity) among individuals in the same subpopulation determined by the STRUCTURE analysis using 31,807 SNPs.

| **Subpopulation groups** | ***F*_ST_** | **Heterozygosity** | **Number of genotypes** | **Distribution of genotype origin** |
| --- | --- | --- | --- | --- |
| G1 | 0.9575 | 0.0240 | 11 | 4 China (36.36 %); 1 Mexico (9%); 1 North Korea (9%); 5 South Korea (45.45%) |
| G2 | 0.4478 | 0.2128 | 53 | 2 China (3.77%); 2 Georgia (3.77%); 27 Japan (50.94%); 2 North Korea (3.77%); 1 Russia (1.88%); 19 South Korea (35.84 %); 1 Taiwan (1.88%) |
| G3 | 0.4892 | 0.1892 | 86 | 3 China (3.48%); 1 India (1.16%); 2 Korea (2.32%); 1 North Korea (1.16%); 1 Romania (1.16%); 78 South Korea (90.69%) |
| G4 | 0.9068 | 0.0408 | 37 | 37 South Korea (100%) |
| G5 | 0.3697 | 0.3134 | 48 | 35 China (72.91%); 4 Georgia (8.33 %); 2 Japan (4.16%); 2 North Korea (4.16%); 1 Russia (2.08%); 3 South Korea (6.25); 1 Taiwan (2.08) |
| G6 | 0.9859 | 0.0025 | 8 | 8 South Korea (100%) |
| G7 | 0.4548 | 0.2113 | 39 | 1 China (2.56%); 1 Japan (2.56%); 1 Korea (2.56%); 4 North Korea (10.25%); 32 South Korea (82.05%) |
| G8 | 0.5287 | 0.2401 | 6 | 1 China (16.66%); 5 South Korea (83.33%) |
